# Heterogeneous treatment effect of immune checkpoint inhibitors by pretreatment prognosis in randomized controlled trials

**DOI:** 10.1093/jncics/pkaf127

**Published:** 2026-01-07

**Authors:** Lee X Li, Adel Shahnam, Ganessan Kichenadasse, Lewis Murray, Richard Woodman, Ahmad Y Abuhelwa, Natansh D Modi, Andrew Rowland, Ashley M Hopkins, Michael J Sorich

**Affiliations:** College of Medicine and Public Health, Flinders Health and Medical Research Institute, Flinders University, Adelaide, Australia; Department of Medical Oncology, Peter McCallum Cancer Centre, Melbourne, Australia; College of Medicine and Public Health, Flinders Health and Medical Research Institute, Flinders University, Adelaide, Australia; Department of Medical Oncology, Flinders Medical Centre, Flinders Centre for Innovation in Cancer, Flinders University, Adelaide, Australia; Department of Radiation Oncology, Royal Adelaide Hospital, Adelaide, Australia; College of Medicine and Public Health, Flinders Health and Medical Research Institute, Flinders University, Adelaide, Australia; Department of Pharmacy Practice and Pharmacotherapeutics, College of Pharmacy, University of Sharjah, Sharjah, United Arab Emirates; Clinical and Health Sciences, University of South Australia, Adelaide, Australia; College of Medicine and Public Health, Flinders Health and Medical Research Institute, Flinders University, Adelaide, Australia; College of Medicine and Public Health, Flinders Health and Medical Research Institute, Flinders University, Adelaide, Australia; College of Medicine and Public Health, Flinders Health and Medical Research Institute, Flinders University, Adelaide, Australia

## Abstract

**Background:**

Treatment response to immune checkpoint inhibitors varies considerably, a phenomenon known as heterogeneity of treatment effect. Heterogeneity of treatment effect is explored via one-variable-at-a-time subgroup analyses in randomized controlled trials (RCTs), however, this method has limitations, which the risk-modeling approach seeks to address.

**Methods:**

Applying the risk-modeling approach, individual patient data from 10 RCTs (6 supporting US Food and Drug Administration’s atezolizumab label: OAK, IMpower130, IMpower150, IMpower133, IMbrave150, IMspire150; 4 unlabeled indications: IMpower131, IMpower132, IMmotion151, and IMvigor211) were analyzed by an extreme gradient-boosting algorithm to predict pretreatment prognosis for overall survival. The predicted risk scores were evaluated as efficacy modifiers categorically (high-, intermediate-, low-risk groups) and continuously in Cox models with treatment-by-risk-group interaction terms. Sensitivity and exploratory analyses investigated absolute and meta-analyzed treatment effect and compared the results with established prognostic tools and treatment effect predictors. Statistical significance tests are 2-sided.

**Results:**

Among the 10 RCTs (*n *= 7053), one trial (IMvigor211) showed statistically significant heterogeneity of treatment effect by pretreatment prognosis across all evaluations (risk groups, risk scores, sensitivity analyses: *P* < .001). Among other trials, no statistically significant heterogeneity of treatment effect was detected (risk group and risk score analysis interaction test: OAK *P* = .61 and *P* = .77; IMpower130 *P* = .13 and *P* = .52; IMpower131 *P* = .21 and *P* = .02; IMpower150 *P* = .14 and *P* = .36; IMpower133 *P* = .38 and *P* = .12; IMbrave150 *P* = .15 and *P* = .08; IMspire150 *P* = .24 and *P* = .6; IMpower132 *P* = .15 and *P* = .81; IMmotion151 *P* = .48 and *P* = .21, respectively).

**Conclusions:**

The risk-modeling approach showed no clear link between pretreatment prognosis and immune checkpoint inhibitor efficacy in most RCTs, particularly those supporting atezolizumab’s Food and Drug Administration label. In IMvigor211, patients with better pretreatment prognosis were more likely to benefit from atezolizumab treatment for platinum-refractory metastatic urothelial carcinoma.

## Introduction

Physicians often face uncertainty about how individual patients will respond to a new treatment, despite reported outcomes provided by the best evidence from randomized controlled trials (RCTs). This uncertainty is even more relevant and important for high-cost, high-stakes therapeutics such as the immune checkpoint inhibitors. Over the past decade, immune checkpoint inhibitors, such as atezolizumab, have transformed the landscape of cancer treatment.^1^ However, significant heterogeneity of treatment effect exists; whereas some patients experience positive and durable treatment responses, many derive limited efficacy.^2^ Heterogeneity of treatment effect presents challenges to clinicians, such as when communicating prognosis, and to patients, whose hopes and uncertainties influence important decisions about care and future planning.^3-5^ Thus, a nuanced understanding of the relationship between pretreatment prognosis and treatment outcome in oncology clinical trials is pertinent.

Although the conventional one-variable-at-a-time subgroup analysis in RCTs offers insights into heterogeneity of treatment effect,^6^ it is limited by methodological issues of multiplicity and low statistical power, making it vulnerable to committing false-positive and false-negative errors.^7^ Additionally, because patients do not present with an isolated feature, the conventional subgroup analyses offer little translational relevance. To address these limitations, the risk-modeling approach has been proposed for implementation alongside, or even prioritized over,^8^ the conventional subgroup analysis.^9-11^ The 2-step risk-modeling approach first predicts pretreatment prognosis using multiple patient characteristics then assesses the role of this predicted prognosis in modifying treatment efficacy.^10^ A simulation study suggested that this approach may increase power compared with the conventional subgroup analysis.^12^ By condensing multiple patient characteristics into a single score, it enhances translational relevance while addressing the issues of statistical power and multiplicity.

Therefore, our study aimed to apply the risk-modeling approach using internally developed prognostic models to estimate and explore pretreatment prognosis as a predictor of heterogeneity of treatment effect among 10 atezolizumab RCTs for the treatment of advanced non-small cell lung cancer (NSCLC), small cell lung cancer, melanoma, hepatocellular carcinoma, urothelial cancer, and renal-cell carcinoma.

## Methods

### RCTs and study population

This investigation incorporated all phase 3, comparator-controlled, parallel-design clinical studies made available through a data request for atezolizumab trials submitted to Hoffmann-La Roche and Genentech via the Vivli data sharing platform (January 2022). The available data comprised intention-to-treat populations from 6 RCTs supporting the US Food and Drug Administration (FDA) label for atezolizumab^13^ (OAK,^14^ IMpower130,^15^ IMpower150,^16^ IMpower133,^17^ IMbrave150,^18^ and IMspire150)^19^ and 4 on unlabeled indications (IMvigor211,^20^ IMmotion151,^21^ IMpower131,^22^ and IMpower132^23^.) The study protocols have been previously published;^14-23^  Table S1 summarizes key trial characteristics. To align with primary trial analysis, only 1 of 2 active arms of the 3-arm RCTs (IMpower131 and IMpower150) was included, and patients of IMpower130 and IMpower150 with epidermal growth factor receptor or anaplastic lymphoma kinase alterations were excluded.^15^^,^^16^ Lastly, the mainland China cohort in IMbrave150 was unavailable because of health data sharing restrictions.^24^

Secondary analysis of anonymized individual patient data (IPD) was classified as minimal-risk research by the Southern Adelaide Local Health Network and exempted from review. All participants provided written informed consent as documented in published reports. Patients were not involved in the design, conduct, analysis, reporting, or dissemination of this investigation.

### Predictors and outcome

Predictors of overall survival, including the National Cancer Institute comorbidity indexes (Supplementary Methods),^25^^,^^26^ were selected based on data availability and prior literature^27-36^ and entered into models without further selection. Table S2 lists trialwise prognostic predictors and their specifications. For both the prognostic model and the treatment effect estimations, the outcome of interest was overall survival, defined across trials as the time from random assignment or first dose to the last follow-up or death from any cause.

### Statistical analysis

**Figure 1.**
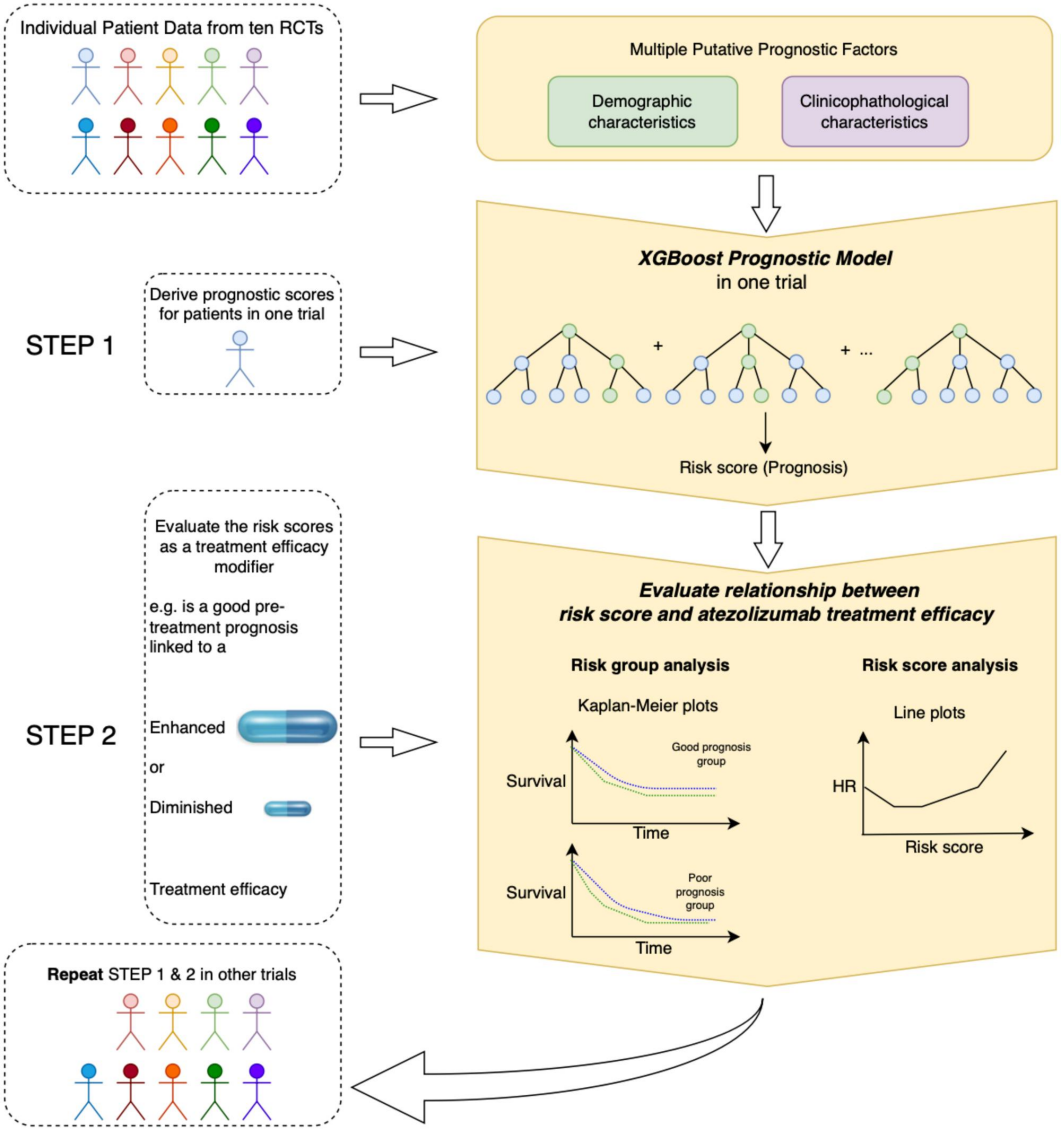
A visual overview of the risk-modeling approach. Abbreviations: HR = hazard ratio; RTC = randomized controlled trial

Figure 1 provides a visual overview of the risk-modeling approach. Median follow-up was estimated by reverse Kaplan–Meier method. All analyses were performed using R (v4.2.3). All statistical tests were 2-sided, with *P* values less than .05 considered statistically significant.

#### Prognostic prediction model

For each RCT, an internal prognostic model predicting overall survival was constructed using an extreme gradient-boosting algorithm (XGBoost). These models did not include treatment assignment as a variable to avoid biases while preserving performance.^37^ XGBoost was chosen for its efficiency, capability to handle missing data, and ability to capture covariate interactions and nonlinear relationships,^38^ thereby minimizing model assumptions and biases from missing data treatment (eg, complete-case analysis or multiple imputation). The latter feature also enabled inclusion of all IPD in the subsequent efficacy analysis, maximizing statistical power. Table S3 describes the XGBoost algorithm (preprocessing, hyperparameter tuning ranges). The hyperparameters were tuned using all data in each RCT with respect to Harrell concordance index (C index) using Bayesian optimization^39^ (Supplementary Methods). The XGBoost models estimated a risk score (linear predictor) for each patient, with higher scores indicating poorer prognosis. The rationale for using the risk scores as potential treatment effect modifiers is explained in Supplementary Methods. As recommended,^10^^,^^40^ median-to-mean risk ratios, measuring risk score skewness, are described in Supplementary Methods.

#### Evaluation of prognostic prediction model

Internal model evaluation assessed discrimination (C index) and calibration (calibration plots). For generalizability, evaluation measures were derived from 10 independent holdout test datasets in a cross-validation procedure, specifically a 10-by-5-fold nested cross-validation as detailed in Supplementary Methods. Model overfitting was assessed by comparing apparent and cross-validated C indexes. To add model interpretability and face validity, variable importance and contribution were explored via Shapley Additive exPlanations (SHAP) analysis using fastSHAP approximations (fastSHAP package v0.1.1^41^) and were visualized in beeswarm charts (shapviz package v0.10.2^42^) Where heterogeneity of treatment effect was identified, uncertainties in variable importance in the relevant model were assessed using 1000 bootstrapped samples to derive 95% confidence intervals (CIs) for variable ranks.

#### Treatment efficacy estimation

Within each RCT, patients were stratified into tertile groups (high, intermediate, and low risk) based on the predicted risk scores. Atezolizumab treatment efficacy (atezolizumab vs control) in these risk groups was estimated on a relative scale in hazard ratios (HRs) via an unstratified Cox proportional hazards model (Coxph, via Survival package v3.5-3), which included a treatment indicator term, a risk group term, and a treatment-by-risk-group interaction term to capture heterogeneity of treatment effect. The Coxph model was not stratified by random assignment factors to align with the conventional subgroup analyses.^14-23^ The interaction term was tested using likelihood ratio test. Overall survival by treatment arm and tertile risk group was visualized in Kaplan–Meier plots, complemented by forest plots displaying groupwise treatment efficacy. Confirmation of the risk group analysis was sought through risk score analysis, where treatment efficacy was estimated across the continuous predicted risk scores modeled flexibly using restricted cubic splines. Similar to the risk group analysis, potential heterogeneity of treatment effect was modeled in Coxph with a treatment-by-risk-score interaction term and tested by likelihood ratio tests. Treatment efficacy estimates were presented in line plots against risk score percentiles. Further details on the risk score analysis including restricted cubic spline specifications and line plot interpretations are provided in Supplementary Methods.

Two sensitivity analyses were performed. First, the absolute treatment effect, measured by the difference in restricted mean survival times between the atezolizumab and the control arms, was estimated in a spline-based flexible parametric survival regression (flexsurv, via flexsurv package v2.3.2)^43^ with a treatment-by-risk-score interaction term. The absolute treatment effect analysis is described in Supplementary Methods. Second, to further address the limited power in subgroup analysis, 2 meta-analyses were conducted to respectively aggregate treatment effect by risk group and risk score across NSCLC trials using methods by Riley and colleagues^44^ (Supplementary Methods).

#### Exploratory analysis

To compare the risk-modeling approach with established prognostic tools and treatment effect predictors, 2 exploratory risk group analyses were conducted. First, in IMmotion151, treatment effect was estimated in risk groups stratified by the Memorial Sloan Kettering Cancer Centre (MSKCC) staging system for renal-cell carcinoma.^45^ Second, in the trial(s) where heterogeneity of treatment effect was identified, treatment effect was estimated in programmed cell death ligand 1 (PD-L1) expression groups. Additionally, the discriminatory performances (apparent, optimism corrected C indexes with 95% confidence intervals derived from internal validation using 1000 bootstrapped samples) of MSKCC and PD-L1 groups were also calculated using Somer D_xy_ estimates from coxph models (rms package v6.3-0).^46^

## Results

### Study population characteristics

All available IPD from the intention-to-treat population of 10 trials (*n* = 7053) were included in the analysis, comprising 1225 from OAK; 686 from IMpower130; 696 from IMpower150; 402 from IMpower133; 423 from IMbrave150; 514 from IMspire150; 683 from IMpower131; 578 from IMpower132; 915 from IMmotion151; and 931 from IMvigor211. Table S4 summarizes the analysis cohort characteristics, including median follow-up, event rates, and overall treatment effect estimates. Table S5 summarizes patient pretreatment demographic and clinicopathologic characteristics by arm in each RCT.

### Prognostic model and model evaluation

**Figure 2.**
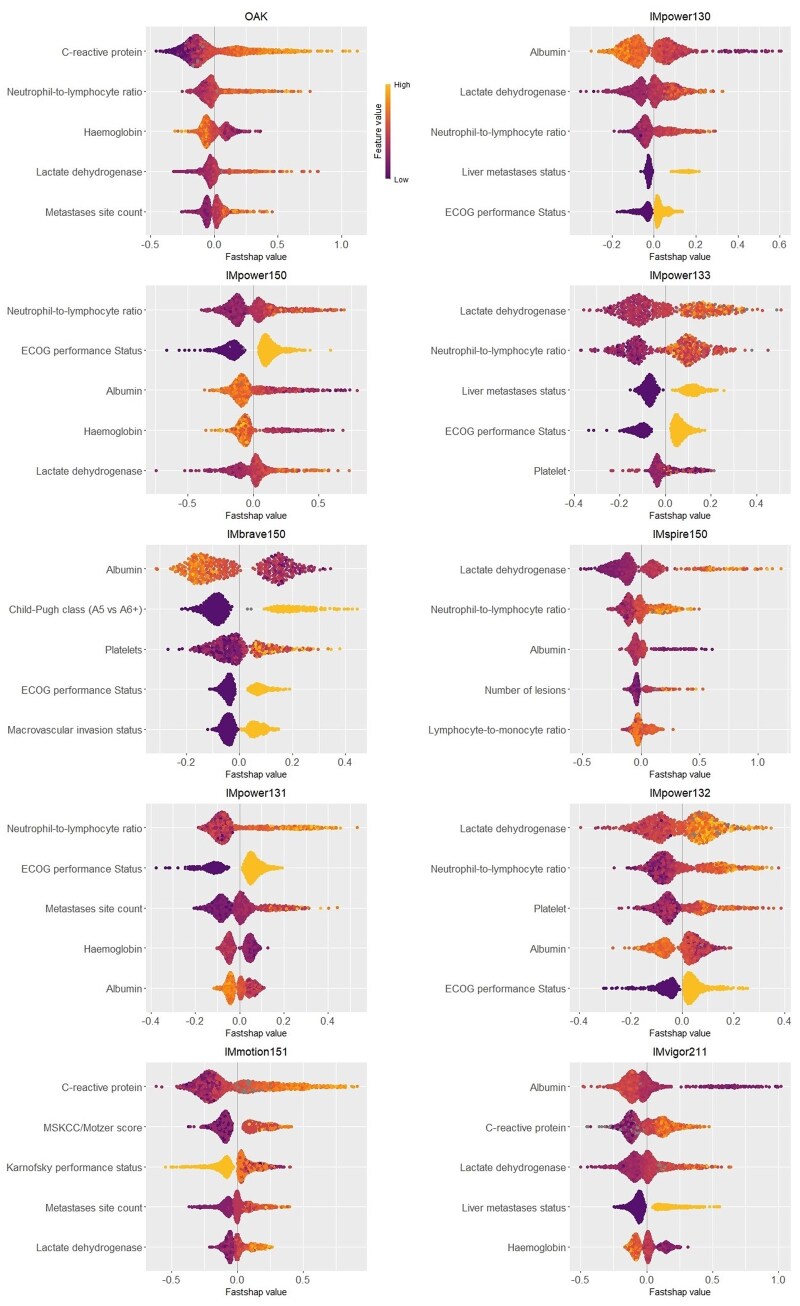
Beeswarm charts from the Shapley Additive Explanations (SHAP) analysis of the prognostic models showing the top 5 variables in descending order of importance. Each **dot** represents an individual’s fastSHAP value for a given variable, showing the variable’s contribution to the model’s prediction for the individual. **Dot color** represents individual’s minimum-maximum scaled variable value, with missing values colored in **gray**. Greater absolute fastSHAP values signify a greater contribution to the model prediction, with negative values indicating lower predicted risk (better prognosis) and positive values indicating higher predicted risk (worse prognosis). Abbreviations: ECOG = Eastern Cooperative Oncology Group; MSKCC = Memorial Sloan Kettering Cancer Center.

Across trials, predicted risk scores were consistently right-skewed except IMpower133 (Figure S1). A moderate to fair model discrimination was observed (cross-validated median C index = 0.66, interquartile range [IQR] = 0.62-0.79) (Table S6). No severe miscalibration was observed across all trials (Figure S2). As expected given small to medium sample RCT sizes,^47^ some models showed small degrees of model overfitting and optimism, reflected in differences between apparent and cross-validated C index (minimum = 0.06, median = 0.11, maximum = 0.14). Despite this, cross-validated calibration (Figure S2) showed no systematic miscalibration, supporting model reliability.^47^ Notably, IMvigor211 exhibited only a small C index difference of 0.08. Figure 2 shows the 5 most important variables and their model prediction contributions in trialwise SHAP beeswarm charts. Lactate dehydrogenase, albumin, neutrophil-to-lymphocyte ratio, and C-reactive protein were commonly rated as the most important variable. In IMvigor211, albumin (median rank = 1, 95% CI = 1 to 2), C-reactive protein (median rank = 2, 95% CI = 1 to 3), lactate dehydrogenase (median rank = 3, 95% CI = 2 to 3), hemoglobin (median rank = 5, 95% CI = 4 to 6), and liver metastasis status (median rank = 5, 95% CI = 4 to 6) were the top 5 variables across the 1000 bootstrapped samples (Table S7). These largely align with prior reports,^48^ except for performance status (Eastern Cooperative Oncology Group Performance Status), which ranked lower (median rank = 12, 95% CI = 10 to 14; best rank = 4) likely reflecting the narrow Eastern Cooperative Oncology Group Performance Status range (0 or 1) in this highly selected cohort.

### Heterogeneity of treatment efficacy

**Figure 3.**
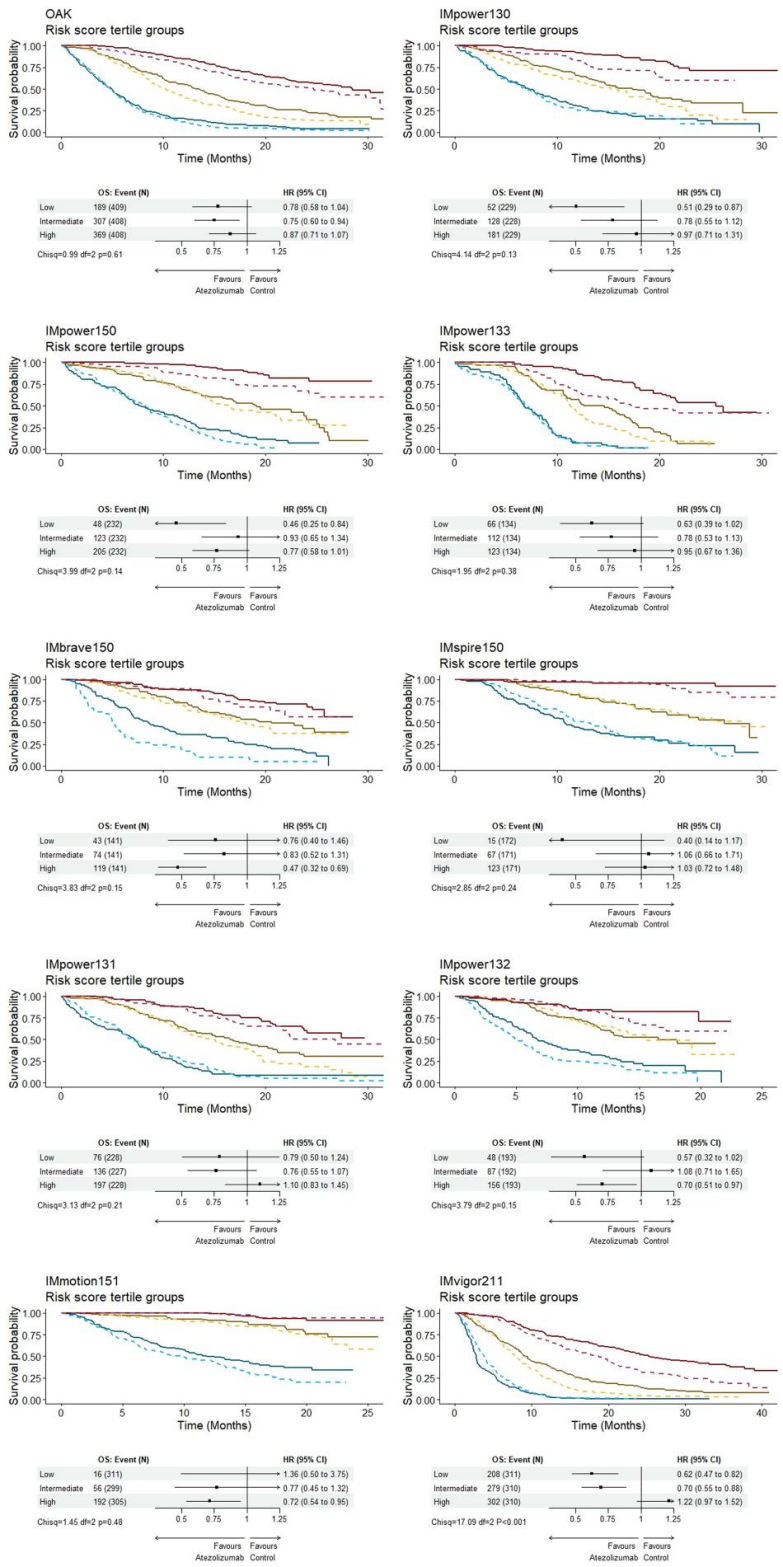
Overall survival in Kaplan–Meier plots and hazard ratios in forest plots for the high-, intermediate-, and low-risk groups. **Solid lines**: atezolizumab-based treatment arms. **Dashed lines**: control arms. **Red**: low-risk group. **Yellow**: intermediate-risk group. **Blue**: high-risk group. Abbreviations: CI = confidence interval; HR = hazard ratio; OS = overall survival.

**Figure 4.**
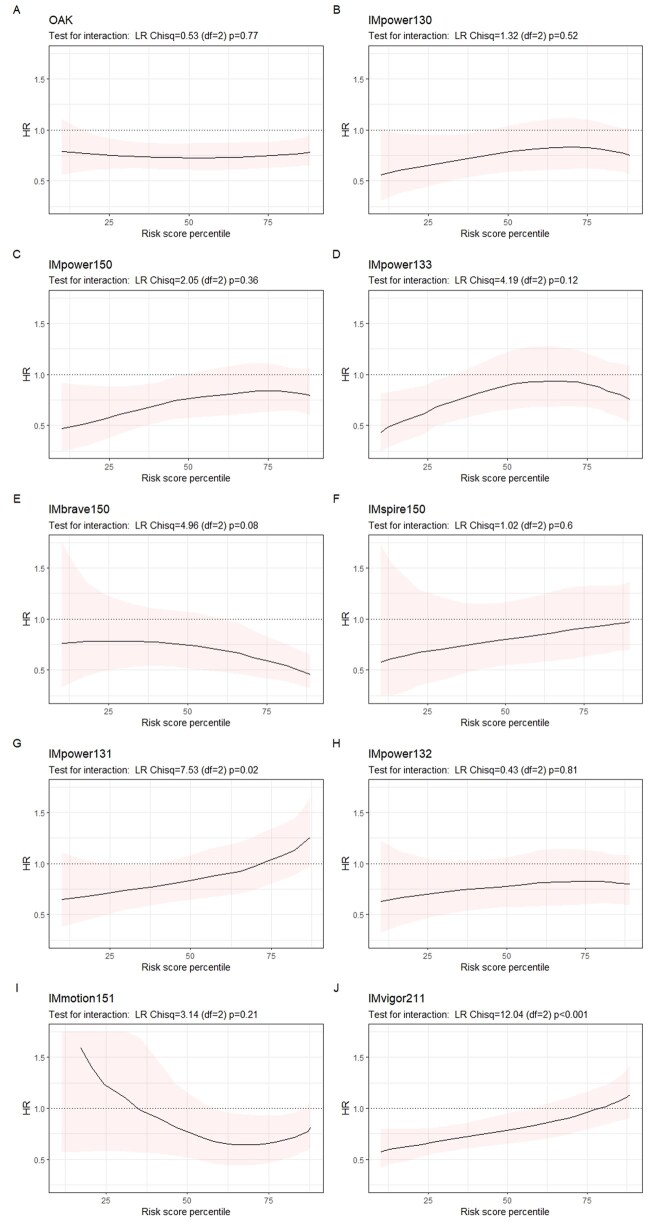
Line plots of treatment efficacy (atezolizumab vs control) across continuous risk score percentiles. Abbreviations: HR = hazard ratio; LR = likelihood ratio.

Figures 3 and 4 present the results from the risk group and risk score analysis. Overall, no consistent trend of heterogeneity of treatment effect was observed across trials (Figures 3 and 4). From the risk group analysis, most trials showed no evidence for heterogeneity of treatment effect (Figure 3; Oak: *P* = .61; IMpower130: *P* = .13; IMpower150: *P* = .14; IMpower133: *P* = .38; IMbrave150: *P* = .15; IMspire150: *P* = .24; IMpower131: *P* = .21; IMpower132: *P* = .15; IMmotion151: *P* = .48), except for IMvigor211, which displayed statistically significant treatment efficacy variations (Figure 3; IMvigor211: *P* < .001). This is demonstrated by a greater separation between the atezolizumab and control arms in the in the low-risk (better prognosis) group (HR = 0.62, 95% CI = 0.47 to 0.82), indicating greater treatment efficacy compared with other risk groups, particularly the high-risk (poorer prognosis) group (HR = 1.22, 95% CI = 0.97 to 1.52).

Analysis with continuous risk scores further supported the presence of heterogeneity of treatment effect in IMvigor211, both by the interaction test (Figure 4J; χ^2^ = 12.04, *df* = 2, *P* < .001) and the line plot, where treatment effect line and its confidence intervals crossed the reference line (HR = 1) into the region indicating treatment benefit (HR < 1), suggesting greater certainty of treatment benefits among patients with lower risk compared with those with higher risk in this trial. Additionally, this pattern of treatment efficacy variation was also observed in IMpower131 in the risk score analysis (Figure 4G; χ^2^  = 7.53, *df *= 2, *P* = .02).

Sensitivity analyses demonstrated consistent findings. The absolute treatment efficacy estimates (control restricted mean survival times minus atezolizumab restricted mean survival times) aligned with the relative scale results (Figure S3 vs Figure 4). Notably, statistically significant interaction terms consistently supported heterogeneity of treatment effect in IMvigor211 (Figure S3J; *P* < .001). Furthermore, the meta-analyses of NSCLC RCTs similarly showed a lack of evidence for heterogeneity of treatment effect across the risk groups (Figure S4, forest plot: *P* = .16) and scores (Figure S4, line plot: *P* = .36).

### Exploratory analysis

Similar to the risk-modeling approach, patients identified by MSKCC as high risk had greater certainty of atezolizumab treatment benefit, and this efficacy variation was not statistically significant (Figure S5, interaction test: *P* = .21). A key difference between the MSKCC and the risk-modeling approach is their discriminatory performance: an apparent C index of 0.66 from the former (Figure S5; 95% CI = 0.63 to 0.68, optimism-corrected C index = 0.66) compared with 0.84 for the internal XGBoost model (Table S6; cross-validated C index = 0.79).

In alignment with the primary IMvigor211 analysis,^20^^,^^49^ the trial cohort was stratified into 3 groups based on PD-L1 expression levels on tumor-infiltrating immune cells (IC groups: IC0, IC1, and IC3). The PD-L1 IC groups did not demonstrate treatment effect variations (Figure S6, interaction test: *P* = .93), nor did they show prognostic values (C index = 0.52, 95% CI = 0.51 to 0.56).

## Discussion

This study explored heterogeneity of treatment effect by pretreatment prognosis across 10 atezolizumab RCTs by applying internally developed XGBoost models to risk stratify patients. This risk-modeling approach did not provide insights into heterogeneity of treatment effect in most trials, including the 6 RCTs supporting the current atezolizumab FDA label.

The risk-modeling approach has been widely described outside of oncology, with several successful implementations,^50-52^ particularly in cardiovascular medicine.^40^^,^^53-57^ Although no prior cancer trials explicitly implemented this approach, mapping treatment outcome by prognosis is not novel in oncology. Such risk-stratified subgroup analyses are implemented among trials on cancers with established prognostic tools. For example, the Barcelona Clinic Liver Cancer staging system and MSKCC risk groups were explored in the subgroup analysis of IMbrave150^18^ and IMmotion151.^21^ However, not all cancers have established applicable prognostic tools with good discriminatory performances in the trial population of interest. For instance, in the IMbrave150 cohort, only stages B and C of the Barcelona Clinic Liver Cancer staging system were applicable. In IMmotion151, the discriminatory performance of MSKCC was limited, as demonstrated in our exploratory analysis and previous validation studies.^58^ The risk-modeling approach also allows integration of contemporary prognostic factors and provides continuous risk predictions for nuanced analysis and interpretation.

Although earlier risk-modeling investigations suggested a prevalent presence of heterogeneity of treatment effect on the absolute scale^40^^,^^52^^,^^54-57^ and reported a positive correlation between treatment benefit and prognosis in cardiovascular trials,^40^^,^^53-57^ our analysis did not confirm these findings. Despite maximizing analytical power by including all available IPD and meta-analyzing multiple NSCLC RCTs, heterogeneity of treatment effect based on prognosis was undetected in most trials, suggesting that treatment effect variation by pretreatment prognosis may be rare and/or difficult to detect. Other than the recognized sample size issues,^7^ several trial characteristics can also influence the power to detect heterogeneity of treatment effect, including eligibility criteria that affect cohort risk homogeneity and features of intervention and/or comparator that influence the main and interaction treatment effect. For example, the efficacy profile of atezolizumab may be systematically different in evaluations against controls sharing a common treatment backbone (atezolizumab used alongside comparator, eg, IMpower trials) vs controls built on different regimens^59^ (atezolizumab substituting comparator, eg, IMvigor211, OAK, IMmotion151, IMbrave150). Further, the effect of prognosis on treatment efficacy in cancer RCTs may be subtler and harder to detect, given that participants are preselected based on established prognostic factors such as cancer stage and performance status, leading to more homogeneous trial cohorts. Beyond cohort homogeneity, unpredictable disease processes or insufficiently robust prognostic models may also constrain the detection of heterogeneity of treatment effect by baseline risk.^60^ These considerations bear directly on our investigation, given the complexity of advanced cancers and the intricate relationship between prognosis and cancer treatment outcomes—shaped not only by disease-specific factors but also by broader, unmeasured determinants and influences.^3^

Nevertheless, we found strong evidence of heterogeneity of treatment effect in one RCT (IMvigor211) that remains robust with a Bonferroni-adjusted alpha of 0.005 (*n* = 10). This finding is consistent with a prior report of reduced benefit among high-risk patients identified by an external prognostic model.^61^ By contrast, no heterogeneity of treatment effect was detected across IMvigor211 PD-L1 expression groups, despite PD-L1 being an established biomarker for immune checkpoint inhibitor efficacy. Further, in IMpower131, moderate evidence of heterogeneity of treatment effect was observed in the risk score and absolute treatment effect analyses but not in the risk group analysis or sensitivity meta-analyses, calling for further investigation in other NSCLC trials.

We do, however, emphasize caution in interpreting our findings, acknowledging the concerns with RCT post hoc analysis, particularly in null trials. For the only positive heterogeneity of treatment effect signal identified, in IMvigor211, a biological rationale remains unclear. If genuine, given the nature of the comparator arm, the heterogeneity may reflect variability in the atezolizumab effect, the chemotherapy control treatments, or both. However, robust subgroup investigations, such as risk modeling, have the potential to make null RCTs more constructive by shifting attention toward valid hypothesis-generating post hoc investigations, encouraging clinically meaningful and transparent RCT designs and conduct.^62-64^ Additionally, because null trials often investigated unregistered indications unlikely to be guideline-recommended, understanding these trials beyond the summary results may provide insights into clinically justified, albeit controversial, off-label prescribing in oncology.^65^ Further, even in the absence of any heterogeneity of treatment effect findings, prognostic models can still be valuable, enabling absolute treatment benefit evaluation from a given relative effect estimate. These considerations underscore the potential of a risk-modeling approach to advance RCT analyses beyond traditional methods that have stagnated for decades.^66^

Our study has several limitations. First, despite our best effort, the included prognostic predictors were not exhaustive, as some key genetic biomarkers (eg, tumor mutational burden, microsatellite instability and mismatch repair status) were unavailable or incomplete. Second, absolute treatment effects (restricted mean survival time differences) were estimated at a fixed horizon, which may not suit trials with different follow-up times. The best practice of exploring multiple horizons^67^ was unfortunately beyond our scope. Last, the homogeneity of clinical trial cohorts may limit the generalizability of our findings to real-world populations. Nevertheless, our analysis was robust because of the high-quality clinical trial data and the advanced machine-learning and analytical methods applied.

Future research directions include developing high-performance prognostic models, potentially integrating multimodal data. As with all post hoc analyses, our findings from IMvigor211 require independent confirmation in similar immune checkpoint inhibitor trials. Further, the fundamental limitation of low statistical power in subgroup analyses may be mitigated by large-scale IPD meta-analyses. Finally, other methods for heterogeneity of treatment effect investigation should be explored, such as the metalearners that directly estimate treatment effect.^68^

Applying the risk-modeling approach, our analysis did not uncover statistically significant relationships between pretreatment prognosis and immune checkpoint inhibitors efficacy in trials supporting the atezolizumab FDA label. However, statistically significant heterogeneity of treatment effect was observed in IMvigor211, indicating that patients with favorable pretreatment prognoses were more likely to experience greater benefits from atezolizumab monotherapy for platinum-refractory metastatic urothelial carcinoma.

## Supplementary Material

pkaf127_Supplementary_Data

## Data Availability

This publication is based on research using data from Roche that has been made available through Vivli, Inc. Vivli has not contributed to or approved and is not in any way responsible for the contents of this publication.
