# Supplementary material for: Heterogeneous treatment effect of immune checkpoint inhibitors by pretreatment prognosis in randomized controlled trials
Source: JNCI Cancer Spectr. 2026 Jan 7;10(2):pkaf127. doi: 10.1093/jncics/pkaf127 (PMC13126119; doi:10.1093/jncics/pkaf127)

Supplementary Methods

1. *National Cancer Institute Comorbidity Index*

NCI comorbidity indexes were derived using medical history information available as free-text description rather than ICD code as the latter was unavailable. Weights applied to medical conditions are sourced from a technical report by Stedman and colleagues (M.R. et al.; National Cancer Institute). Due to a lack of reliable temporal information regarding the medical history in relation to study enrolment, the categories of historical and acute myocardial infarction (MI) are combined and the larger weight of 1.14 associated with acute MI was applied. Weight for history of MI in the originally reported NCI comorbidity indexes is 1.08 (M.R. et al.; National Cancer Institute).

Condition	Weight
Myocardial infarction<br>Including historical and acute myocardial infarction, and a broader term of ischaemic heart disease	1.14
Congestive heart failure	1.91
Peripheral vascular disease	1.30
Cerebrovascular disease	1.32
Chronic pulmonary disease	1.69
Dementia	2.06
Paralysis	1.49
Diabetes (any type and severity)	1.34
Chronic kidney disease	1.60
Liver disease (any severity)	2.09
Peptic ulcer disease	1.08
Connective tissue disease (Rheumatologic disease)	1.25
Acquired immunodeficiency syndrome	1.79

2. *Methods on Bayesian optimisation*

Bayesian optimisation (BO) was chosen as the hyperparameter tuning method due to its demonstrated superior performance and efficiency compared to random search or grid search (Bergstra & Bengio, 2012; Turner et al., 2021).

BO was specified in the current analysis as simple Kriging regression with a Gaussian process and Matérn 5/2 kernel that uses Broyden-Fletcher-Goldfarb-Shanno (BFGS) algorithm as an optimiser to find the optimal kernel parameters. Expected improvement function was used as the acquisition function, which was optimised by the DIRECT algorithm with a termination criterion defined as improvement of less

than 0.01 for 50 iterations. Efficient Global Optimisation algorithm was used as the loop function during the BO.

This BO procedure was setup in mlr3 by the following code:

```
loop=mlr_loop_functions$get("bayesopt_ego")
sur=srlrn(lrn("regr.km", covtype="matern5_2",
          optim.method="BFGS",
          control=list(trace=F)))
acq=acqf("ei")
acq_opt=acqo(opt("nloptr", algorithm="NLOPT_GN_ORIG_DIRECT"),
         terminator=trm("stagnation",iters=50, threshold=0.01))

tuner = tnr("mbo",
        loop_function=loop,
        surrogate=sur,
        acq_function=acq,
        acq_optimizer=acq_opt)
```

3. *Rationale for using risk scores as a potential treatment effect modifier*

Prognostic scores, traditionally derived from weighted sum of model coefficients in regression methods and presently estimated directly from the XGBoost algorithm in our study, represent event risks (prognosis) described through a combination of patient characteristics. They have been used for RCT subgroup analysis (Goligher et al., 2023; Kent et al., 2016; Pocock et al., 2001; Sormani et al., 2023), and construction of clinical risk prediction tools, such as nomogram, risk scale, risk scores (Harrell, 2015; Steyerberg, 2019).

Differences in study participants' pre-treatment prognosis or risks for the clinical outcome of interest are a recognised potential source of clinically important HTE (Kent et al., 2010; Kent et al., 2018; Rothwell, 2005). Variables that implicitly or explicitly measure prognosis are routinely applied for RCT stratification and/or subgrouping, such as performance status, Bellmunt score for urothelial cancer, and Memorial Sloan Kettering Cancer Centre score for renal cell carcinoma. Moreover, pretreatment prognosis has been proposed as a prioritised subgrouping variable over other patient characteristics in RCT subgroup analysis (Kent et al., 2018). Because patient baseline risks may not be evenly distributed (Kent et al., 2016), as illustrated in Supplementary Figure 1 of the present study, resulting in under- or over-representation of low or high risk patients, and given that prognosis and prognostication are important considerations in

cancer care, the risk-modelling approach emerges as a rational first-step toward understanding HTE.

4. Risk score skewness measurement - median-to-mean risk ratios (MMRRs)

To enable consistent comparison across clinical trials, predicted risk scores derived from each trial dataset were min–max scaled, mapping the minimum and maximum values in each trial to zero and one, respectively, while preserving the underlying distributional shape. Skewness of the risk scores were evaluated by the mean-to-median risk ratio (MMRR), defined as the ratio of the average predicted risk to the median predicted risk within each trial population. An MMRR value of one indicates a symmetric (approximately normal) distribution, whereas values less than one reflect right-skewed distributions. The more pronounced the right-skewness, the greater the potential for disparities between the median and the mean risk in the trial population, which could potentially lead to discrepancies between the reported summary treatment effect (the average) and what was most likely experienced by a trial participant (the median) (Kent & Hayward, 2007; Kent et al., 2016).

5. Evaluation of XGBoost prognostic models (discrimination and calibration evaluation)

The XGBoost prognostic model for each RCT was evaluated in terms of discrimination and calibration via Harrell's C-index and calibration plots. Model evaluation was performed in ten independent holdout test datasets during a cross-validation procedure. Specifically, a ten-by-five-fold nested cross-validation (nCV) procedure was carried out, with the inner five folds for hyperparameter tuning (via random split 5-fold CV), and the outer ten folds for model evaluation. For each trial, the C-indexes derived across the holdout evaluation datasets (folds) were aggregated by averaging. Cross-validated (aggregated) calibration plots were derived via the model calibration evaluations (Austin et al., 2020) assessed at 24 months, as described below.

Calibration plot construction

We adopted the hazard regression (HARE) approach (Austin et al., 2020) for the estimation of observed event probabilities, with the predicted event probabilities estimated at 24 months from the XGBoost prognostic models. For each RCT, a cross-validated ICI plot indicating overall calibration was constructed by pooling event probability predictions from all ten outer evaluation cohorts and fitting a single HARE for the entire analysis cohort in the trial. The accuracy of

prediction – calibration – is visualised by plotting the observed probabilities (y-axis) against the predicted probabilities (x-axis) in a line-plot with the reference line of identity at 45 degrees from the axis.

6. *Methods on risk score analysis - estimation of treatment effect by risk score*

In each RTC, a Cox proportional-hazard model (Coxph) was constructed incorporating a treatment indicator term, risk score term, and a treatment-by-risk-score interaction term, followed by estimation of unstratified hazard ratios (HRs) (atezolizumab vs control) at risk scores corresponding to the 2.5 to 97.5 percentile of the risk score distribution in the RCT. The interaction terms were tested using the likelihood ratio (LR) test for nested models (models with and without the interaction term). In all Coxph models, the risk scores were flexibly modelled by restricted cubic splines (RCS) with three knots placed at default positions.

Inference and interpretation of line plots

The LR tests for interaction terms complement the graphical displays of treatment effect against risk scores (in percentiles) in the line plots as described in the main report. A straight treatment effect line parallel to the reference line (HR=1 and RMST difference = 0) indicates a lack of HTE, whereas a non-constant line (increasing, decreasing, or curved), particularly if the crossing of the reference line is evident, signals potential differential treatment effects.

7. *Methods on sensitivity analysis – risk score analysis on absolute treatment effect*

Extending above risk score analysis, in each RCT, a sensitivity analysis was carried out to explore treatment effect measured on an absolute scale, quantified by the difference in restricted mean survival times (RMSTs) between the atezolizumab and control arms. This analysis involved modelling with spline-based flexible parametric survival (flexsurv) regression (Jackson, 2016), specified with a treatment indicator term, risk score term, and a treatment-by-risk-score interaction term with the baseline survival and the risk scores both modelled by RCS with three default knots to capture any potential non-linear effects. RMSTs at 24 months were predicted from the flexsurv regression for each arm at risk scores corresponding to the 2.5 to 97.5 percentile of the risk score distribution in the RCT.

The absolute treatment effect was derived by the difference in the predicted RMSTs between the arms. Specifically, the difference was calculated as RMST[control arm] minus RMST[atezolizumab-based treatment arm] to ensure that the absolute treatment effect is interpreted in the same direction as the relative treatment effect in HRs - a negative value indicates benefits from atezolizumab-based treatment. This facilitates comparisons between the absolute and relative treatment effect estimates.

8. Methods on sensitivity analysis – meta-analysis of treatment effect in NSCLC RCTs

Aggregated treatment efficacy estimates were derived using two-stage individual patient data multivariate meta-analyses as outlined by Riley and colleagues (Riley et al., 2020).

The first stage of this analysis had already been performed during the treatment effect estimation, as described in the methods section of the main report, via the Cox proportional-hazard model (Coxph) in each trial with the following terms: treatment assignment, risk score or risk group, and risk (score or group)-by-treatment interaction. In the case of risk score analysis, the restricted cubic splines were applied with 3 knots placed at the same (default) positions in terms of risk score percentile across all trials.

For the second stage of the meta-analysis, two random-effect models were constructed, respectively estimating the aggregated treatment efficacy across the risk groups and the risk scores. Both models assumed an unstructured covariance structure and estimated by the restricted maximum likelihood method implemented via the mixmeta package (v1.2.0) in R. Confidence intervals of the aggregated treatment effect were derived by Wald-based estimation.

For risk group analysis, all coefficient estimates from the Coxph and their corresponding variance-covariance matrices from stage one were entered in the random effect model. Inclusion of all coefficients enables treatment effect estimation in individual risk groups, and, thus, visualisation of the aggregated treatment effect in a forest plot in a similar fashion as the individual trial analysis.

For risk score analysis, serving as a confirmation of the presence or absence of any differential treatment efficacy across the risk scores, only the interaction term coefficients from stage one Coxph were entered in the random-effect model, with the median risk score (50% risk score percentile) as the reference point.

References:

Austin, P. C., Harrell, F. E., Jr., & van Klaveren, D. (2020). Graphical calibration curves and the integrated calibration index (ICI) for survival models. *Stat Med*, *39*(21), 2714-2742. https://doi.org/10.1002/sim.8570

Bergstra, J., & Bengio, Y. (2012). Random search for hyperparameter optimisation. *Journal of machine learning research 13*, 281-305.

FDA. (2024). *U.S. Food and Drug Administration - FDA label for atezolizumab* https://www.accessdata.fda.gov/drugsatfda_docs/label/2024/761034s053lbl.pdf

Finn Richard, S., Qin, S., Ikeda, M., Galle Peter, R., Ducreux, M., Kim, T.-Y., Kudo, M., Breder, V., Merle, P., Kaseb Ahmed, O., Li, D., Verret, W., Xu, D.-Z., Hernandez, S., Liu, J., Huang, C., Mulla, S., Wang, Y., Lim Ho, Y.,...Cheng, A.-L. (2020). Atezolizumab plus Bevacizumab in Unresectable Hepatocellular Carcinoma. *New England Journal of Medicine*, *382*(20), 1894-1905. https://doi.org/10.1056/NEJMoa1915745

Goligher, E. C., Lawler, P. R., Jensen, T. P., Talisa, V., Berry, L. R., Lorenzi, E., McVerry, B. J., Chang, C. H., Leifer, E., Bradbury, C., Berger, J., Hunt, B. J., Castellucci, L. A., Kornblith, L. Z., Gordon, A. C., McArthur, C., Webb, S., Hochman, J., Neal, M. D.,...Angus, D. C. (2023). Heterogeneous Treatment Effects of Therapeutic-Dose Heparin in Patients Hospitalized for COVID-19. *Jama*, *329*(13), 1066-1077. https://doi.org/10.1001/jama.2023.3651

Gutzmer, R., Stroyakovskiy, D., Gogas, H., Robert, C., Lewis, K., Protsenko, S., Pereira, R. P., Eigentler, T., Rutkowski, P., Demidov, L., Manikhas, G. M., Yan, Y., Huang, K. C., Uyei, A., McNally, V., McArthur, G. A., & Ascierto, P. A. (2020). Atezolizumab, vemurafenib, and cobimetinib as first-line treatment for unresectable advanced BRAF(V600) mutation-positive melanoma (IMspire150): primary analysis of the randomised, double-blind, placebo-controlled, phase 3 trial. *Lancet*, *395*(10240), 1835-1844. https://doi.org/10.1016/s0140-6736(20)30934-x

Harrell, J. F. E. (2015). *Regression Modeling Strategies With Applications to Linear Models, Logistic and Ordinal Regression, and Survival Analysis* (2nd ed. 2015. ed.). Springer International Publishing. https://doi.org/10.1007/978-3-319-19425-7

Horn, L., Mansfield Aaron, S., Szczęsna, A., Havel, L., Krzakowski, M., Hochmair Maximilian, J., Huemer, F., Losonczy, G., Johnson Melissa, L., Nishio, M., Reck, M., Mok, T., Lam, S., Shames David, S., Liu, J., Ding, B., Lopez-Chavez, A., Kabbinavar, F., Lin, W.,...Liu Stephen, V. (2018). First-Line Atezolizumab plus Chemotherapy in Extensive-Stage Small-Cell Lung Cancer. *New England Journal of Medicine*, *379*(23), 2220-2229. https://doi.org/10.1056/NEJMoa1809064

Jackson, C. H. (2016). flexsurv: A Platform for Parametric Survival Modeling in R. *J Stat Softw*, *70*. https://doi.org/10.18637/jss.v070.i08

Jotte, R., Cappuzzo, F., Vynnychenko, I., Stroyakovskiy, D., Rodríguez-Abreu, D., Hussein, M., Soo, R., Conter, H. J., Kozuki, T., Huang, K.-C., Graupner, V., Sun, S. W., Hoang, T., Jessop, H., McCleland, M., Ballinger, M., Sandler, A., & Socinski, M. A. (2020). Atezolizumab in Combination With Carboplatin and Nab-Paclitaxel in Advanced Squamous NSCLC (IMpower131): Results From a Randomized Phase III Trial. *Journal of thoracic oncology*, *15*(8), 1351-1360. https://doi.org/10.1016/j.jtho.2020.03.028

Kent, D. M., & Hayward, R. A. (2007). Limitations of Applying Summary Results of Clinical Trials to Individual Patients: The Need for Risk Stratification. *JAMA : the journal of the American Medical Association*, *298*(10), 1209-1212. https://doi.org/10.1001/jama.298.10.1209

Kent, D. M., Nelson, J., Dahabreh, I. J., Rothwell, P. M., Altman, D. G., & Hayward, R. A. (2016). Risk and treatment effect heterogeneity: re-analysis of individual participant data from 32 large clinical trials. *International journal of epidemiology*, *45*(6), 2075-2088. https://doi.org/10.1093/ije/dyw118

Kent, D. M., Rothwell, P. M., Ioannidis, J. P. A., Altman, D. G., & Hayward, R. A. (2010). Assessing and reporting heterogeneity in treatment effects in clinical trials: a proposal. *Current controlled trials in cardiovascular medicine*, *11*(1), 85-85. https://doi.org/10.1186/1745-6215-11-85

Kent, D. M., Steyerberg, E., & van Klaveren, D. (2018). Personalized evidence based medicine: predictive approaches to heterogeneous treatment effects. *BMJ*, *363*, k4245. https://doi.org/10.1136/bmj.k4245

M.R., S., P., D.-R., J.L., W., C.N., K., & A., M. The Impact of Different SEER-Medicare Claims-based Comorbidity Indexes on

Predicting Non-cancer Mortality for Cancer Patients. https://healthcaredelivery.cancer.gov/seermedicare/considerations/comorbidity-report.pdf
Motzer, R. J., Powles, T., Atkins, M. B., Escudier, B., McDermott, D. F., Alekseev, B. Y., Lee, J.-L., Suarez, C., Stroyakovskiy, D., De Giorgi, U., Donskov, F., Mellado, B., Banchereau, R., Hamidi, H., Khan, O., Craine, V., Huseni, M., Flinn, N., Dubey, S., & Rini, B. I. (2022). Final Overall Survival and Molecular Analysis in IMmotion151, a Phase 3 Trial Comparing Atezolizumab Plus Bevacizumab vs Sunitinib in Patients With Previously Untreated Metastatic Renal Cell Carcinoma. *JAMA Oncology*, *8*(2), 275-280. https://doi.org/10.1001/jamaoncol.2021.5981
National Cancer Institute. NCI Comorbidity Index Overview. https://healthcaredelivery.cancer.gov/seermedicare/considerations/comorbidity.html
Nishio, M., Barlesi, F., West, H., Ball, S., Bordoni, R., Cobo, M., Longeras, P. D., Goldschmidt, J., Novello, S., Orlandi, F., Sanborn, R. E., Szalai, Z., Ursol, G., Mendus, D., Wang, L., Wen, X., McCleland, M., Hoang, T., Phan, S., & Socinski, M. A. (2021). Atezolizumab Plus Chemotherapy for First-Line Treatment of Nonsquamous NSCLC: Results From the Randomized Phase 3 IMpower132 Trial. *Journal of thoracic oncology*, *16*(4), 653-664. https://doi.org/10.1016/j.jtho.2020.11.025
Pocock, S. J., McCormack, V., Gueyffier, F., Boutitie, F., Fagard, R. H., & Boissel, J. P. (2001). A score for predicting risk of death from cardiovascular disease in adults with raised blood pressure, based on individual patient data from randomised controlled trials. *BMJ*, *323*(7304), 75-81. https://doi.org/10.1136/bmj.323.7304.75
Powles, T., Durán, I., van der Heijden, M. S., Loriot, Y., Vogelzang, N. J., De Giorgi, U., Oudard, S., Retz, M. M., Castellano, D., Bamias, A., Fléchon, A., Gravis, G., Hussain, S., Takano, T., Leng, N., Kadel, E. E., 3rd, Banchereau, R., Hegde, P. S., Mariathasan, S.,...Ravaud, A. (2018). Atezolizumab versus chemotherapy in patients with platinum-treated locally advanced or metastatic urothelial carcinoma (IMvigor211): a multicentre, open-label, phase 3 randomised controlled trial. *Lancet*, *391*(10122), 748-757. https://doi.org/10.1016/s0140-6736(17)33297-x
Riley, R. D., Debray, T. P. A., Fisher, D., Hattle, M., Marlin, N., Hoogland, J., Gueyffier, F., Staessen, J. A., Wang, J., Moons, K. G. M., Reitsma, J. B., & Ensor, J. (2020). Individual participant data meta-analysis to examine interactions between treatment effect and participant-level covariates: Statistical recommendations for conduct and planning. *Stat Med*, *39*(15), 2115-2137. https://doi.org/10.1002/sim.8516
Rothwell, P. M. (2005). Treating individuals 2. Subgroup analysis in randomised controlled trials: importance, indications, and interpretation. *Lancet*, *365*(9454), 176-186. https://doi.org/10.1016/s0140-6736(05)17709-5
Sormani, M. P., Chataway, J., Kent, D. M., & Marrie, R. A. (2023). Assessing heterogeneity of treatment effect in multiple sclerosis trials. *Mult Scler*, *29*(9), 1158-1161. https://doi.org/10.1177/13524585231189673
Steyerberg, E. (2019). *Clinical Prediction Models. A practical approach to development, validation, and updating (2 Ed.)* (2 ed.). Springer Nature. https://doi.org/https://doi.org/10.1007/978-3-030-16399-0
Turner, R., Eriksson, D., McCourt, M. J., Kiili, J., Laaksonen, E., Xu, Z., & Guyon, I. M. (2021). Bayesian Optimization is Superior to Random Search for Machine Learning Hyperparameter Tuning: Analysis of the Black-Box Optimization Challenge 2020. Neural Information Processing Systems,
West, H., McCleod, M., Hussein, M., Morabito, A., Rittmeyer, A., Conter, H. J., Kopp, H. G., Daniel, D., McCune, S., Mekhail, T., Zer, A., Reinmuth, N., Sadiq, A., Sandler, A., Lin, W., Ochi Lohmann, T., Archer, V., Wang, L., Kowanetz, M., & Cappuzzo, F. (2019). Atezolizumab in combination with carboplatin plus nab-paclitaxel chemotherapy compared with chemotherapy alone as first-line treatment for metastatic non-squamous non-small-cell lung cancer (IMpower130): a multicentre, randomised, open-label, phase 3 trial. *Lancet Oncol*, *20*(7), 924-937. https://doi.org/10.1016/s1470-2045(19)30167-6

Supplementary Tables

Table S1: *Characteristics of the ten randomised controlled trials. Information in this table was sourced from previously published data except Data cut-off dates, which refer to the cut-off dates of the analysis data.*

RCT	NCT	Data cut-off	FDA label	Cancer / population	Design	Treatment line	Active Arm (N**)	Control Arm (N**)	Cross-over	Published OS Analysis population
OAK (Rittmeyer et al., 2017)	NCT02008227	23/01/2017	Yes	Locally advanced or metastatic NSCLC (squamous or non-squamous) whose disease progressed during or following a platinum-containing regimen	Randomised (1:1), open-label	Second or later-line	Atezolizumab monotherapy (613)	Docetaxel (612)	Not allowed	First 850 of 1225 enrolled ITT population and PD-L1 expression population
IMpower130 (West et al., 2019)	NCT02367781	15/3/2018	Yes	Metastatic chemotherapy-naïve non-squamous NSCLC with (prior EGFR or ALK kinase inhibitor allowed)	Randomised (2:1), open-label	First-line	Atezolizumab + carboplatin + nab-paclitaxel (483)	Carboplatin + nab-paclitaxel (240)	Cross-over occurred in up to 60% of chemotherapy group	ITT wild type ($EGFR^{wt}$ and ALK^{wt})
IMpower150 (Socinski Mark et al., 2018)	NCT02366143	22/01/2018	Yes	Metastatic chemotherapy-naïve non-squamous NSCLC with (prior EGFR or ALK kinase inhibitor allowed)	Randomised (1:1:1), open-label	First-line	Atezolizumab + bevacizumab + carboplatin + paclitaxel (400)<br>Atezolizumab + carboplatin + paclitaxel (402)	Bevacizumab + carboplatin + paclitaxel (400)	Not allowed	ITT wild type ($EGFR^{wt}$ and ALK^{wt})<br>ABCP vs BCP
IMpower133 (Horn et al., 2018)	NCT02763579	24/01/2019	Yes	Extensive-stage SCLC	Randomised (2:1), double-blind	First-line	Atezolizumab + carboplatin + etoposide (201)	Carboplatin + etoposide (202)	Not planned	ITT
IMbrave150 (Finn Richard et al., 2020)	NCT03434379	31/08/2020	Yes	Systemic therapy-naïve locally advanced unresectable and/or metastatic HCC	Randomised (2:1), open-label	First-line systemic therapy	Atezolizumab + bevacizumab (336)	Sorafenib (165)	Not planned	ITT
IMspire150 (Gutzmer et al., 2020)	NCT02908672	11/09/2019	Yes	Untreated stage IV or unresectable stage IIIc $BRAF^{v600}$ mutation-positive melanoma	Randomised (1:1) double-blind	First-line	Atezolizumab + vemurafenib + cobimetinib (256)	Vemurafenib + cobimetinib (258)	Not planned	ITT (OS was not a primary efficacy outcome)

IMpower131 (Jotte et al., 2020)	NCT02367794	20/04/2018	No	Stage IV squamous NSCLC	Randomised (1:1:1), open-label	First-line	Atezolizumab + carboplatin + paclitaxel (338) (ACP)<br>Atezolizumab + carboplatin + nab-paclitaxel (343) (ACnP)	Carboplatin + nab-paclitaxel (340) (CnP)	Not allowed	ITT ACnP vs CnP
IMpower132 (Nishio et al., 2021)	NCT02657434	22/05/2018	No	Stage IV non-squamous NSCLC	Randomised (1:1), open-label	First-line	Atezolizumab + carbo/cisplatin + pemetrexed (292)	carbo/cisplatin + pemetrexed (286)	Not allowed	ITT
IMmotion151 (Rini et al., 2019)	NCT02420821	29/09/2017	No	Metastatic renal cell carcinoma	Randomised (1:1), open-label	First-line (mRCC)	Atezolizumab + Bevacizumab (454)	Sunitinib (461)	Not planned	ITT (OS was not a primary efficacy outcome)
IMvigor211 (Powles et al., 2018)	NCT02302807	08/11/2018	No	Metastatic urothelial carcinoma progressed after platinum-based chemotherapy	Randomised (1:1), open-label	Previously treated	Atezolizumab (467)	Chemotherapy (464)	Not planned	Sequentially testing in IC2/3 PDL1, IC1/2/3 and ITT cohorts

Stratified HRs: by PD-L1 expression in tumor infiltrating immune cells, the number of prior chemotherapy regimens, and histology

Stratified by sex and PD-L1 tumor expression on tumor cells (TC) and tumor infiltrating cells (IC)

Stratified by sex, presence of liver metastases, and PD-L1 expression status on TC and IC

* Stratified by sex and ECOG performance status

** Stratified by geographic region (Asia excluding Japan vs. rest of world), macrovascular invasion and/or extrahepatic spread (presence vs. absence), and baseline AFP (<400 vs. ≥400 ng/mL)

*** Stratified by baseline LDH

† Stratified but detail not reported, assuming the same stratification factor for randomisation: sex, smoking status (never versus current or former), Eastern Cooperative Oncology Group performance status (0 versus 1), and chemotherapy regimen (cisplatin versus carboplatin)

†† Stratified by presence of liver metastasis (yes/no); tumor PD-L1 status (IC0 vsIC1/2/3); and the Memorial Sloan Kettering Cancer Center/Motzer risk score (0 [favorable], 1-2 [intermediate], and≥3 [poor]).

††† Stratified by PD-L1 expression, chemotherapy type, liver metastases, and number of prognostic factors (none vs one, two, or three—defined as time from previous chemotherapy <3 months, ECOG performance status ≥1, and haemoglobin<10 g/dL)

List of published clinical study reports (cited in supplementary table 1):

Finn Richard, S., Qin, S., Ikeda, M., Galle Peter, R., Ducreux, M., Kim, T.-Y., Kudo, M., Breder, V., Merle, P., Kaseb Ahmed, O., Li, D., Verret, W., Xu, D.-Z., Hernandez, S., Liu, J., Huang, C., Mulla, S., Wang, Y., Lim Ho, Y.,...Cheng, A.-L. (2020). Atezolizumab plus Bevacizumab in Unresectable Hepatocellular Carcinoma. *New England Journal of Medicine*, *382*(20), 1894-1905. https://doi.org/10.1056/NEJMoa1915745

Gutzmer, R., Stroyakovskiy, D., Gogas, H., Robert, C., Lewis, K., Protsenko, S., Pereira, R. P., Eigentler, T., Rutkowski, P., Demidov, L., Manikhas, G. M., Yan, Y., Huang, K. C., Uyei, A., McNally, V., McArthur, G. A., & Ascierto, P. A. (2020). Atezolizumab, vemurafenib, and cobimetinib as first-line treatment for unresectable advanced BRAF(V600) mutation-positive melanoma (IMspire150): primary analysis of the randomised, double-blind, placebo-controlled, phase 3 trial. *Lancet*, *395*(10240), 1835-1844. https://doi.org/10.1016/s0140-6736(20)30934-x

Horn, L., Mansfield Aaron, S., Szczęsna, A., Havel, L., Krzakowski, M., Hochmair Maximilian, J., Huemer, F., Losonczy, G., Johnson Melissa, L., Nishio, M., Reck, M., Mok, T., Lam, S., Shames David, S., Liu, J., Ding, B., Lopez-Chavez, A., Kabbinavar, F., Lin, W.,...Liu Stephen, V. (2018). First-Line Atezolizumab plus Chemotherapy in Extensive-Stage Small-Cell Lung Cancer. *New England Journal of Medicine*, *379*(23), 2220-2229. https://doi.org/10.1056/NEJMoa1809064

Jotte, R., Cappuzzo, F., Vynnychenko, I., Stroyakovskiy, D., Rodríguez-Abreu, D., Hussein, M., Soo, R., Conter, H. J., Kozuki, T., Huang, K.-C., Graupner, V., Sun, S. W., Hoang, T., Jessop, H., McCleland, M., Ballinger, M., Sandler, A., & Socinski, M. A. (2020). Atezolizumab in Combination With Carboplatin and Nab-Paclitaxel in Advanced Squamous NSCLC (IMpower131): Results From a Randomized Phase III Trial. *Journal of thoracic oncology*, *15*(8), 1351-1360. https://doi.org/10.1016/j.jtho.2020.03.028

Nishio, M., Barlesi, F., West, H., Ball, S., Bordoni, R., Cobo, M., Longeras, P. D., Goldschmidt, J., Novello, S., Orlandi, F., Sanborn, R. E., Szalai, Z., Ursol, G., Mendus, D., Wang, L., Wen, X., McCleland, M., Hoang, T., Phan, S., & Socinski, M. A. (2021). Atezolizumab Plus Chemotherapy for First-Line Treatment of Nonsquamous NSCLC: Results From the Randomized Phase 3 IMpower132 Trial. *Journal of thoracic oncology*, *16*(4), 653-664. https://doi.org/10.1016/j.jtho.2020.11.025

Powles, T., Durán, I., van der Heijden, M. S., Loriot, Y., Vogelzang, N. J., De Giorgi, U., Oudard, S., Retz, M. M., Castellano, D., Bamias, A., Fléchon, A., Gravis, G., Hussain, S., Takano, T., Leng, N., Kadel, E. E., 3rd, Banchereau, R., Hegde, P. S., Mariathasan, S.,...Ravaud, A. (2018). Atezolizumab versus chemotherapy in patients with platinum-treated locally advanced or metastatic urothelial carcinoma (IMvigor211): a multicentre, open-label, phase 3 randomised controlled trial. *Lancet*, *391*(10122), 748-757. https://doi.org/10.1016/s0140-6736(17)33297-x

Rini, B. I., Powles, T., Atkins, M. B., Escudier, B., McDermott, D. F., Suarez, C., Bracarda, S., Stadler, W. M., Donskov, F., Lee, J. L., Hawkins, R., Ravaud, A., Alekseev, B., Staehler, M., Uemura, M., De Giorgi, U., Mellado, B., Porta, C., Melichar, B.,...Motzer, R. J. (2019). Atezolizumab plus bevacizumab versus sunitinib in patients with previously untreated metastatic renal cell carcinoma (IMmotion151): a multicentre, open-label, phase 3, randomised controlled trial. *The Lancet (British edition)*, *393*(10189), 2404-2415. https://doi.org/10.1016/S0140-6736(19)30723-8

Rittmeyer, A., Barlesi, F., Waterkamp, D., Park, K., Ciardiello, F., von Pawel, J., Gadgeel, S. M., Hida, T., Kowalski, D. M., Dols, M. C., Cortinovis, D. L., Leach, J., Polikoff, J., Barrios, C., Kabbinavar, F., Frontera, O. A., De Marinis, F., Turna, H., Lee, J.-S.,...Gandara, D. R. (2017). Atezolizumab versus docetaxel in patients with previously treated non-small-cell lung cancer (OAK): a phase 3, open-label, multicentre randomised controlled trial. *The Lancet*, *389*(10066), 255-265. https://doi.org/10.1016/S0140-6736(16)32517-X

Socinski Mark, A., Jotte Robert, M., Cappuzzo, F., Orlandi, F., Stroyakovskiy, D., Nogami, N., Rodríguez-Abreu, D., Moro-Sibilot, D., Thomas Christian, A., Barlesi, F., Finley, G., Kelsch, C., Lee, A., Coleman, S., Deng, Y., Shen, Y., Kowanetz, M., Lopez-Chavez, A., Sandler, A., & Reck, M. (2018). Atezolizumab for First-Line Treatment of Metastatic Nonsquamous NSCLC. *New England Journal of Medicine*, *378*(24), 2288-2301. https://doi.org/10.1056/NEJMoa1716948

West, H., McCleod, M., Hussein, M., Morabito, A., Rittmeyer, A., Conter, H. J., Kopp, H. G., Daniel, D., McCune, S., Mekhail, T., Zer, A., Reinmuth, N., Sadiq, A., Sandler, A., Lin, W., Ochi Lohmann, T., Archer, V., Wang, L., Kowanetz, M., & Cappuzzo, F. (2019). Atezolizumab in combination with carboplatin plus nab-paclitaxel chemotherapy compared with chemotherapy alone as first-line treatment for metastatic non-squamous non-small-cell lung cancer (IMpower130): a multicentre, randomised, open-label, phase 3 trial. *Lancet Oncol*, *20*(7), 924-937. https://doi.org/10.1016/s1470-2045(19)30167-6

Table S2: *Pre-treatment prognostic factors included in XGBoost prognostic models*

RCT	Predictors included
OAK	Age (numeric), sex (male or female), ECOGPS (0 or ≤ 1), metastatic site count, histology (squamous vs non-squamous), smoking history (current, past, or never), liver metastasis status (present or absent), bone metastasis status (present or absent), EGFR/ALK (present or wild type/missing), haemoglobin levels, LDH (logarithmically transformed), ALB, Creatinine (logarithmically transformed), PD-L1 IC group (numeric, 0-3), PD-L1 TC group (numeric, 0-3), platelet (logarithmically transformed), CRP (logarithmically transformed), NLR (logarithmically transformed), NCI comorbidity index[#] (numeric), baseline CNS metastasis status (present or absent)
IMpower130, IMpower131, IMpower150	Age (numeric), sex (male or female), ECOGPS (0 or ≤ 1), metastatic site count, smoking history (current, past, or never), liver metastasis status (present or absent), bone metastasis status (present or absent), EGFR/ALK (present or wild type/missing), haemoglobin levels, LDH (logarithmically transformed), ALB, Creatinine (logarithmically transformed), PD-L1 IC scores, PD-L1 TC scores, platelet (logarithmically transformed), NLR (logarithmically transformed), NCI comorbidity index[#] (numeric), baseline CNS metastasis status (present or absent)
IMpower132	Age (numeric), sex (male or female), ECOGPS (0 or ≤ 1), metastatic site count, smoking history (current, past, or never), liver metastasis status (present or absent), bone metastasis status (present or absent), EGFR/ALK (present or wild type/missing), haemoglobin levels, LDH (logarithmically transformed), ALB, Creatinine (logarithmically transformed), platelet (logarithmically transformed), NLR (logarithmically transformed), NCI comorbidity index[#] (numeric), baseline CNS metastasis status (present or absent)
IMpower133	Age (numeric), sex (male or female), ECOGPS (0 or ≤ 1), metastatic site count, smoking history (current, past, or never), NLR (logarithmically transformed), liver metastasis status (present or absent), bone metastasis status (present or absent), LDH (logarithmically transformed), ALB, Creatinine, platelet (logarithmically transformed), NCI comorbidity index[#] (numeric), baseline CNS metastasis status (present or absent
IMbrave150	Age (numeric), sex (male or female), ECOGPS (0 or ≤ 1), metastatic site count, varices (present or absent), macrovascular invasion (present or absent), extra-hepatic spread (present or absent), tumour burden occupy >= 50% of liver (present or absent), prior local therapy (yes or no), Child-Pugh class group (class A5 or class A6 and above), Barcelona liver cancer stage (stage A/B or stage C), ALB, Creatinine, platelet, NCI comorbidity index[#] (numeric), HCC caused by HBV or HCV (Yes or No)
IMspire150	Age (numeric), sex (male or female), ECOGPS (0 or ≤ 1), liver metastasis status (present or absent), stage-distant metastasis group (M0/M1A, M1B, or M1C), metastasis site count (numeric, 1-4), SLD, histology (superficial spreading, nodular, or other), BRAF mutation subtype (V600E, V600K, other/unknown), lesion number count group(1-2, 3-4, or 5+), ALB, LDH (logarithmically transformed), NLR (logarithmically transformed), haemoglobin, PLR (logarithmically transformed), LMR (logarithmically transformed), NCI comorbidity index[#] (numeric)
IMvigor211	Age (numeric), sex (male or female), metastatic site counts, PD-L1 IC group (categorical, 0-2), PD-L1 TC group (categorical, 0-3), smoking history (current, former, or never), liver metastasis status (present or absent), bone metastasis status (present or absent), lung metastasis status (present or absent), lymph node metastasis status (present or absent), visceral metastasis status (present or absent), ECOGPS (0 or 1), ALB, creatinine (logarithmically transformed), platelets (logarithmically transformed), CRP (logarithmically transformed), LDH (logarithmically transformed), haemoglobin, NLR (logarithmically transformed), LMR(logarithmically transformed), PLR (logarithmically transformed), NCI comorbidity index[#] (numeric)
IMmotion151	Age (numeric), sex (male or female), metastatic site counts, KARNPS (numeric, 60-100), histology (clear cell, sarcomatoid, or other), MTZER scores (numeric 1-4), liver metastasis status (present or absent), corrected calcium, haemoglobin, platelet (logarithmically transformed), neutrophil (logarithmically transformed), LDH (logarithmically transformed) ALP(logarithmically transformed), CRP (logarithmically transformed), PD-L1 IC scores, NCI comorbidity index[#] (numeric)

NCI comorbidity indexes were derived using medical history information available as free-text description rather than ICD code as the latter was unavailable. ***Supplementary File 1.1*** provides a description of the weights applied to the relevant medical conditions.

Abbreviations:
ALB: albumin
BRAF: B-Raf proto-oncogene
CRP: C-reactive protein
ECOGPS: Eastern Cooperative Oncology Group performance status
EGFR/ALK: epidermal growth factor receptor (EGFR) / echinoderm microtubule-associated protein-like 4 (EML4) - anaplastic lymphoma kinase (ALK) (EML4-ALK) fusion gene rearrangement
HBV: hepatitis B virus infection
HCC: hepatocellular carcinoma
HCV: hepatitis C virus infection
KARNPS: Karnofsky performance status
LDH: lactate dehydrogenase
LMR: lymphocyte-to-monocyte ratio
MTZER: Memorial Sloan Kettering Cancer Centre score, also known as Motzer score
NCI: National Cancer Institute
NLR: neutrophil-to-lymphocyte ratio
PD-L1 IC: infiltrating immune cell PD-L1 expression
PD-L1 TC: tumour cell PD-L1 expression
PLR: platelet-to-lymphocyte ratio
SLD: sum of the longest diameters

Table S3: *Extreme gradient-boosting algorithm (XGBoost) specifications and hyperparameter ranges. For reproducibility, wherever a seed is required, integer 123 was used. The XGBoost models were implemented via mlr3, which also carried out hyperparameter tuning, risk score predictions, and model evaluations.*

**Learner in mlr3**	**Implementation package#**	**Pre-processing‡**	**Model specification* and Hyperparameter tuning range****
surv.xgboost.cox	XGBoost v1.7.8.1	Encode ‡	eta = to_tune(0.001, 0.1)<br>nrounds = to_tune(50, 2000)<br>max_depth = to_tune(1,10)<br>min_child_weight = to_tune(0, 20)<br>gamma = to_tune(0, 10)<br>lambda = to_tune(0, 20)<br>alpha = to_tune(0, 10)<br>colsample_bytree = to_tune(0, 1)

Packages utilised by mlr3 to implement the underlying model/algorithm
‡ Encode refers to one-hot encoding, creating a new column for each factor level
* Specification of model deviating from the default mlr3 learner setup
** Model/Algorithm specified and hyperparameter tuned. Hyperparameter tuning was carried out by Bayesian optimisation implemented in mlr3.

***Table S4**: Analysis cohort count, median follow-up times, event counts, and the overall treatment effect (unstratified HR, atezolizumab vs control) of the included ITT population by trial and by arm*

RCT	Arm	Published N	Analysis N	Median follow-up Months (IQR)	Event (death) Rate Rate (n/N)	Overall (unstratified)* Treatment Effect HR (95% CI)
**OAK**		**1225** (FDA, 2024)	**1225**	**26.3 (22.4 – 28.6)**	**0.71 (865/1225)**	**0.80 (0.70 – 0.91)**
	Atezolizumab	613	613	26.5 (22.6 – 29.0)	0.69 (424/613)	
	Docetaxel	612	612	26.0 (21.9 – 28.5)	0.72 (441/612)	
**IMpower130**		**681** (West et al., 2019)	**686**	**18.6 (15.3 – 23.4)**	**0.53 (361/686)**	**0.81 (0.65 – 1.00)**
	Atezolizumab + Carboplatin + nab-Paclitaxel	453 (ITT/WT)	454 (ITT/WT)	18.5 (15.2 – 23.6)	0.50 (229/454)	
	+ Carboplatin + nab-Paclitaxel	228 (ITT/WT)	232 (ITT/WT)	18.8 (15.4 -23.3)	0.57 (132/232)	
**IMpower150**		**696** (FDA, 2024)	**696**	**19.8 (15.5 – 23.4)**	**0.54 (376/696)**	**0.78 (0.64 – 0.96)**
	Atezolizumab + Bevacizumab + Carboplatin + Paclitaxel	359(ITT/WT)	359 (ITT/WT)	19.8 (15.3 – 23.2)	0.50 (179/359)	
	Bevacizumab + Carboplatin + Paclitaxel	337(ITT/WT)	337 (ITT/WT)	20.0 (15.8 – 24.1)	0.58 (197/337)	
**IMpower133**		**403** (Horn et al., 2018)	**402**	**22.9 (20.7 – 25.2)**	**0.75 (301/402)**	**0.77 (0.61 – 0.96)**
	Atezolizumab + Carboplatin + Etoposide	201	201	23.1 (20.7 – 25.5)	0.71 (142/201)	
	Carboplatin + Etoposide	202	201	22.6 (20.8 – 23.9)	0.79 (159/201)	
**IMbrave150**		**501** (Finn Richard et al., 2020)	**423#**	**22.2 (20.4 – 24.7)**	**0.56 (236/423)**	**0.69 (0.53 – 0.90)**
	Atezolizumab + Bevacizumab	336	279	22.7 (20.7 – 24.9)	0.54 (150/279)	
	Sorafenib	165	144	21.4 (20.0 – 24.0)	0.60 (86/144)	
**IMspire150**		**514** (Gutzmer et al., 2020)	**514**	**22.9 (19.2 – 26.5)**	**0.40 (205/514)**	**0.83 (0.63 – 1.10)**
	Atezolizumab + Vemurafenib + Cobimetinib	256	256	22.8 (19.1 – 26.5)	0.36 (93/256)	
	Vemurafenib + Cobimetinib	258	258	23.2 (19.5 – 26.6)	0.43 (112/258)	
**IMpower131**		**683** (Jotte et al., 2020)	**683**	**19.9 (15.2 – 25.4)**	**0.60 (409/683)**	**0.91 (0.75 – 1.10)**
	Atezolizumab + Carboplatin + nab-Paclitaxel	343	343	21.0 (15.5 – 25.6)	0.59 (202/343)	
	Carboplatin + nab-Paclitaxel	340	340	19.2 (14.8 – 24.6)	0.61 (207/340)	
**IMpower132**		**578** (Nishio et al., 2021)	**578**	**14.8 (13.6 - 17.5)**	**0.50 (291/578)**	**0.80 (0.63 – 1.01)**
	Atezolizumab + Carbo/Cisplatin + Pemetrexed	292	292	15.2 (13.7 – 17.5)	0.47 (137/292)	
	Carbo/Cisplatin + Pemetrexed	286	286	14.5 (13.4 – 17.1)	0.54 (154/286)	
**IMmotion151**		**915** (Motzer et al., 2022)	**915**	**17.1 (14.6 – 20.4)**	**0.29 (264/915)**	**0.83 (0.66 – 1.06)**
	Atezolizumab + Bevacizumab	454	454	17.3 (14.6 – 20.6)	0.27 (123/454)	
	Sunitinib	461	461	16.9 (14.3 – 20.1)	0.31 (141/461)	
**IMvigor211**		**931** (Powles et al., 2018)	**931**	**34.3 (32.2 – 37.6)**	**0.85 (789/931)**	**0.82 (0.71 – 0.94)**
	Atezolizumab	467	467	34.7 (32.4 – 39.6)	0.83 (386/467)	
	Chemotherapy of physician's choice	464	464	34.1 (31.8 – 35.9)	0.87 (403/464)	

Mainland China cohort in IMbrave150 was unavailable due to health data sharing restrictions

* Derived from the analysis cohort

Table S5*: Pre-treatment demographic and clinicopathologic characteristics of the analysis cohort by arm in each randomised controlled trial. ITT: intention-to-treat. WT: wild type.*

OAK ITT population

	**Total**<br>**No. 1,225**	**Atezolizumab**<br>**No. 613**	**Control**<br>**No. 612**
Age	63 (57 - 70)	63 (57 - 69)	64 (57 - 70)
Sex			
Female	467 (38%)	234 (38%)	233 (38%)
Male	758 (62%)	379 (62%)	379 (62%)
ECOG performance status group			
0	455 (37%)	221 (36%)	234 (38%)
1+	770 (63%)	392 (64%)	378 (62%)
Metastasis site count	3 (2 - 4)	3 (2 - 4)	3 (2 - 4)
Haemoglobin	124 (111 - 135)	125 (111 - 136)	123 (110 - 134)
Lactate dehydrogenase			
Median (IQR)	234 (187 - 347)	230 (187 - 348)	238 (187 - 338)
Missing	15 (1%)	7 (1%)	8 (1%)
Albumin			
Median (IQR)	39 (36 - 43)	39 (35 - 42)	40 (36 - 43)
Missing	5 (<1%)	4 (1%)	1 (<1%)
Creatinine			
Median (IQR)	76 (65 - 93)	76 (65 - 92)	76 (65 - 95)
Missing	1 (<1%)	0 (0%)	1 (<1%)
PD-L1 tumour-infiltrating immune cell expression group			
0	595 (49%)	293 (48%)	302 (49%)
1	452 (37%)	237 (39%)	215 (35%)
2	113 (9%)	50 (8%)	63 (10%)
3	55 (4%)	27 (4%)	28 (5%)
Missing	10 (1%)	6 (1%)	4 (1%)
PD-L1 tumour cell expression group			
0	876 (72%)	446 (73%)	430 (70%)
1	102 (8%)	48 (8%)	54 (9%)
2	138 (11%)	65 (11%)	73 (12%)
3	99 (8%)	48 (8%)	51 (8%)
Missing	10 (1%)	6 (1%)	4 (1%)
Platelet levels	264 (213 - 332)	261 (214 - 328)	266 (213 - 337)
C-reactive protein			
Median (IQR)	13 (4 - 40)	14 (4 - 42)	13 (4 - 38)
Missing	46 (4%)	11 (2%)	35 (6%)
Neutrophil-to-lymphocyte ratio			
Median (IQR)	3.5 (2.4 - 5.6)	3.6 (2.4 - 5.8)	3.5 (2.4 - 5.3)
Missing	3 (0.2%)	2 (0.3%)	1 (0.2%)
Histology			
Non-squamous	904 (74%)	452 (74%)	452 (74%)
Squamous	321 (26%)	161 (26%)	160 (26%)
Tobacco history			
Current	190 (16%)	83 (14%)	107 (17%)
Former	827 (68%)	418 (68%)	409 (67%)
Never	208 (17%)	112 (18%)	96 (16%)
Liver metastasis	251 (20%)	126 (21%)	125 (20%)
Bone metastasis	382 (31%)	193 (31%)	189 (31%)
Genetic alteration in EGFR and ALK			
Negative_or_missing	1,116 (91%)	553 (90%)	563 (92%)
Positive	109 (9%)	60 (10%)	49 (8%)
NCI comorbidity score			
Median (IQR)	0 (0 - 2)	0 (0 - 2)	0 (0 - 2)
Missing	46 (4%)	23 (4%)	23 (4%)
CNS metastases at baseline	118 (10%)	52 (8%)	66 (11%)

Data are median (IQR) or number of patients (%).

IMpower130 ITT-WT population

	Total No. 686	Atezolizumab No. 454	Control No. 232
Age	64 (58 - 71)	64 (58 - 71)	65 (58 - 71)
Sex			
Female	280 (41%)	185 (41%)	95 (41%)
Male	406 (59%)	269 (59%)	137 (59%)
ECOG performance status group			
0	280 (41%)	189 (42%)	91 (39%)
1+	405 (59%)	264 (58%)	141 (61%)
Missing	1 (<1%)	1 (<1%)	0 (0%)
Metastasis site count	3 (2 - 3)	3 (2 - 4)	3 (2 - 3)
Haemoglobin			
Median (IQR)	132 (119 - 142)	133 (119 - 141)	132 (118 - 142)
Missing	1 (<1%)	0 (0%)	1 (<1%)
Lactate dehydrogenase			
Median (IQR)	230 (178 - 372)	235 (180 - 372)	220 (175 - 373)
Missing	5 (1%)	3 (1%)	2 (1%)
Albumin			
Median (IQR)	39 (35 - 42)	39 (35 - 42)	38 (36 - 41)
Missing	1 (<1%)	0 (0%)	1 (<1%)
Creatinine	71 (59 - 84)	71 (58 - 84)	71 (62 - 86)
PD-L1 tumour-infiltrating immune cell expression			
Median (IQR)	1.0 (0.1 - 3.0)	1.0 (0.1 - 3.0)	1.0 (0.1 - 3.0)
Missing	1 (0.1%)	0 (0%)	1 (0.4%)
PD-L1 tumour cell expression	0 (0 - 5)	0 (0 - 5)	0 (0 - 8)
Platelet levels			
Median (IQR)	292 (234 - 373)	290 (234 - 373)	296 (234 - 376)
Missing	1 (<1%)	0 (0%)	1 (<1%)
Neutrophil-to-lymphocyte ratio			
Median (IQR)	4.2 (2.9 - 6.2)	4.2 (3.0 - 6.2)	4.2 (2.8 - 6.2)
Missing	1 (0.1%)	0 (0%)	1 (0.4%)
Tobacco history			
Current	144 (21%)	93 (20%)	51 (22%)
Former	475 (69%)	312 (69%)	163 (70%)
Never	67 (10%)	49 (11%)	18 (8%)
Liver metastasis	101 (15%)	70 (15%)	31 (13%)
Bone metastasis	191 (28%)	127 (28%)	64 (28%)
NCI comorbidity score			
Median (IQR)	1.1 (0.0 - 1.7)	1.1 (0.0 - 1.7)	1.2 (0.0 - 1.7)
Missing	12 (1.7%)	9 (2.0%)	3 (1.3%)
CNS metastases at baseline	67 (10%)	40 (9%)	27 (12%)

Data are median (IQR) or number of patients (%).

IMpower150 ITT-WT population

	Total No. 696	Atezolizumab No. 359	Control No. 337
Age	63 (58 - 70)	63 (57 - 70)	63 (58 - 69)
Sex			
Female	269 (39%)	140 (39%)	129 (38%)
Male	427 (61%)	219 (61%)	208 (62%)
ECOG performance status group			
0	283 (41%)	140 (39%)	143 (42%)
1+	407 (58%)	216 (60%)	191 (57%)
Missing	6 (1%)	3 (1%)	3 (1%)
Metastasis site count	2 (1 - 2)	2 (1 - 2)	2 (1 - 2)
Haemoglobin	132 (120 - 143)	131 (120 - 143)	133 (119 - 143)
Lactate dehydrogenase			
Median (IQR)	238 (185 - 342)	237 (183 - 341)	241 (188 - 342)
Missing	7 (1%)	3 (1%)	4 (1%)
Albumin			
Median (IQR)	39 (35 - 42)	39 (34 - 42)	39 (35 - 42)
Missing	4 (1%)	1 (<1%)	3 (1%)
Creatinine	69 (58 - 82)	69 (57 - 82)	69 (59 - 81)
PD-L1 tumour-infiltrating immune cell expression			
Median (IQR)	0.90 (0.00 - 3.00)	0.90 (0.00 - 3.00)	0.90 (0.00 - 3.00)
Missing	1 (0.14%)	0 (0%)	1 (0.30%)
PD-L1 tumour cell expression	0 (0 - 5)	0 (0 - 6)	0 (0 - 5)
Platelet levels	290 (233 - 373)	283 (230 - 378)	296 (234 - 364)
Neutrophil-to-lymphocyte ratio			
Median (IQR)	4.1 (2.7 - 6.6)	4.1 (2.7 - 7.1)	4.1 (2.6 - 6.2)
Missing	1 (0.1%)	1 (0.3%)	0 (0%)
Tobacco history			
Current	167 (24%)	83 (23%)	84 (25%)
Former	420 (60%)	217 (60%)	203 (60%)
Never	109 (16%)	59 (16%)	50 (15%)
Liver metastasis	94 (14%)	47 (13%)	47 (14%)
Bone metastasis	21 (3%)	12 (3%)	9 (3%)
NCI comorbidity score			
Median (IQR)	0 (0 - 2)	0 (0 - 1)	0 (0 - 2)
Missing	25 (4%)	10 (3%)	15 (4%)
CNS metastases at baseline	0 (0%)	0 (0%)	0 (0%)

Data are median (IQR) or number of patients (%).

IMpower133 ITT population

	Total No. 402	Atezolizumab No. 201	Control No. 201
Age			
Median (IQR)	64 (58 - 69)	64 (59 - 69)	64 (58 - 69)
Missing	1 (<1%)	1 (<1%)	0 (0%)
Sex			
Female	142 (35%)	72 (36%)	70 (35%)
Male	260 (65%)	129 (64%)	131 (65%)
ECOG performance status group			
0	140 (35%)	73 (36%)	67 (33%)
1+	262 (65%)	128 (64%)	134 (67%)
Metastasis site count	3 (2 - 4)	3 (2 - 4)	3 (2 - 4)
Lactate dehydrogenase			
Median (IQR)	310 (215 - 492)	308 (215 - 492)	313 (216 - 483)
Missing	12 (3%)	7 (3%)	5 (2%)
Albumin			
Median (IQR)	40 (36 - 43)	40 (36 - 43)	40 (36 - 43)
Missing	11 (3%)	5 (2%)	6 (3%)
Creatinine			
Median (IQR)	71 (59 - 83)	71 (61 - 84)	71 (57 - 82)
Missing	6 (1%)	2 (1%)	4 (2%)
Tobacco history			
Current	149 (37%)	74 (37%)	75 (37%)
Former	242 (60%)	118 (59%)	124 (62%)
Never	11 (3%)	9 (4%)	2 (1%)
Neutrophil-to-lymphocyte ratio			
Median (IQR)	3.4 (2.4 - 5.2)	3.2 (2.4 - 5.4)	3.5 (2.4 - 5.1)
Missing	6 (1.5%)	2 (1.0%)	4 (2.0%)
Liver metastasis	148 (37%)	77 (38%)	71 (35%)
Bone metastasis	83 (21%)	39 (19%)	44 (22%)
Platelet levels			
Median (IQR)	281 (228 - 350)	287 (222 - 353)	279 (232 - 348)
Missing	6 (1%)	2 (1%)	4 (2%)
NCI comorbidity score			
Median (IQR)	1.2 (0.0 - 1.7)	1.3 (0.0 - 1.7)	1.2 (0.0 - 1.7)
Missing	13 (3.2%)	7 (3.5%)	6 (3.0%)
CNS metastases at baseline	35 (9%)	17 (8%)	18 (9%)

Data are median (IQR) or number of patients (%).

IMbrave150 ITT population

	Total<br>No. 423	Atezolizumab<br>No. 279	Control<br>No. 144
Age	66 (59 - 72)	66 (58 - 72)	67 (61 - 72)
Sex			
Female	76 (18%)	51 (18%)	25 (17%)
Male	347 (82%)	228 (82%)	119 (83%)
ECOG performance status group			
0	274 (65%)	179 (64%)	95 (66%)
1+	149 (35%)	100 (36%)	49 (34%)
Albumin	39 (36 - 42)	39 (36 - 43)	39 (35 - 42)
Creatinine	71 (61 - 85)	72 (61 - 84)	71 (61 - 85)
Platelet levels	169 (120 - 224)	173 (120 - 223)	156 (120 - 229)
Varices	119 (28%)	80 (29%)	39 (27%)
Macrovascular invasion	169 (40%)	109 (39%)	60 (42%)
Extra-hepatic spread	253 (60%)	173 (62%)	80 (56%)
Tumour burden occupy ≥50% of liver	28 (7%)	16 (6%)	12 (8%)
Prior local therapy	196 (46%)	124 (44%)	72 (50%)
Child-pugh score group			
A5	295 (70%)	189 (68%)	106 (74%)
A6+	126 (30%)	88 (32%)	38 (26%)
Missing	2 (<1%)	2 (1%)	0 (0%)
Metastatic site count			
0	227 (54%)	145 (52%)	82 (57%)
1	163 (39%)	116 (42%)	47 (33%)
2	31 (7%)	17 (6%)	14 (10%)
3	2 (<1%)	1 (<1%)	1 (1%)
Barcelona Clinic Liver Cancer Stage			
STAGE A/B	79 (19%)	50 (18%)	29 (20%)
STAGE C	344 (81%)	229 (82%)	115 (80%)
NCI comorbidity score			
Median (IQR)	2.1 (0.0 - 3.2)	2.1 (0.0 - 3.2)	2.1 (0.0 - 3.3)
Missing	1 (0.2%)	1 (0.4%)	0 (0%)
HCC caused by HBV/HCV			
Yes	272 (64%)	179 (64%)	93 (65%)
No	110 (26%)	73 (26%)	37 (26%)
Missing	41 (10%)	27 (10%)	14 (10%)

Data are median (IQR) or number of patients (%).

IMspire150 ITT population

	Total No. 514	Atezolizumab No. 256	Control No. 258
Age	54 (43 - 64)	54 (45 - 64)	54 (43 - 64)
Sex			
Female	215 (42%)	106 (41%)	109 (42%)
Male	299 (58%)	150 (59%)	149 (58%)
Liver metastasis			
Yes	155 (30%)	69 (27%)	86 (33%)
No	357 (69%)	187 (73%)	170 (66%)
Missing	2 (<1%)	0 (0%)	2 (1%)
ECOG performance status			
0	393 (76%)	195 (76%)	198 (77%)
1	117 (23%)	61 (24%)	56 (22%)
Missing	4 (1%)	0 (0%)	4 (2%)
Stage-Distant metastasis			
M0/1A	105 (20%)	54 (21%)	51 (20%)
M1B	98 (19%)	56 (22%)	42 (16%)
M1C	308 (60%)	145 (57%)	163 (63%)
Missing	3 (1%)	1 (<1%)	2 (1%)
Number of lesions			
1-2	130 (25%)	72 (28%)	58 (22%)
3-4	173 (34%)	80 (31%)	93 (36%)
5+	208 (40%)	104 (41%)	104 (40%)
Missing	3 (1%)	0 (0%)	3 (1%)
Number of lesions			
Median (IQR)	4 (2 - 6)	4 (2 - 6)	4 (3 - 6)
Missing	3 (1%)	0 (0%)	3 (1%)
Metastasis sites count			
Median (IQR)	2 (2 - 3)	2 (2 - 3)	2 (2 - 3)
Missing	6 (1%)	2 (1%)	4 (2%)
Albumin			
Median (IQR)	43 (39 - 46)	43 (39 - 45)	43 (39 - 46)
Missing	3 (1%)	0 (0%)	3 (1%)
Lactate dehydrogenase	233 (183 - 360)	229 (181 - 349)	240 (184 - 369)
Neutrophil-to-lymphocyte ratio			
Median (IQR)	2.9 (2.0 - 4.2)	2.8 (2.0 - 4.0)	2.9 (2.1 - 4.5)
Missing	3 (0.6%)	0 (0%)	3 (1.2%)
Haemoglobin			
Median (IQR)	137 (126 - 149)	136 (125 - 149)	137 (127 - 148)
Missing	2 (<1%)	0 (0%)	2 (1%)
Sum of the longest diameters			
Median (IQR)	52 (29 - 89)	51 (26 - 87)	54 (31 - 89)
Missing	4 (1%)	1 (<1%)	3 (1%)
Histological subtype at inital diagnosis			
NODULAR	164 (32%)	84 (33%)	80 (31%)
Other	179 (35%)	86 (34%)	93 (36%)
SUPERFICIAL SPREADING	168 (33%)	85 (33%)	83 (32%)
Missing	3 (1%)	1 (<1%)	2 (1%)
BRAF mutation subtype			
Other/Unknown	79 (15%)	32 (12%)	47 (18%)
V600E	379 (74%)	197 (77%)	182 (71%)
V600K	56 (11%)	27 (11%)	29 (11%)
Platelet-to-lymphocyte ratio			
Median (IQR)	164 (125 - 234)	165 (123 - 226)	164 (126 - 246)
Missing	3 (1%)	0 (0%)	3 (1%)
Lymphocyte-to-monocyte ratio			
Median (IQR)	3.0 (2.0 - 4.1)	3.0 (2.0 - 4.1)	3.0 (2.0 - 4.1)
Missing	3 (0.6%)	0 (0%)	3 (1.2%)
NCI comorbidity score			
Median (IQR)	0 (0 - 0)	0 (0 - 0)	0 (0 - 0)
Missing	100 (19%)	42 (16%)	58 (22%)

Data are median (IQR) or number of patients (%).

IMpower131 ITT population

	Total No. 683	Atezolizumab No. 343	Control No. 340
Age	65 (59 - 70)	65 (59 - 70)	65 (60 - 71)
Sex			
Female	126 (18%)	63 (18%)	63 (19%)
Male	557 (82%)	280 (82%)	277 (81%)
ECOG performance status group			
0	225 (33%)	115 (34%)	110 (32%)
1+	456 (67%)	227 (66%)	229 (67%)
Missing	2 (<1%)	1 (<1%)	1 (<1%)
Metastasis site count	4 (3 - 5)	4 (3 - 5)	4 (3 - 5)
Haemoglobin	128 (116 - 139)	128 (115 - 138)	128 (117 - 140)
Lactate dehydrogenase			
Median (IQR)	221 (179 - 320)	220 (177 - 312)	221 (183 - 332)
Missing	12 (2%)	7 (2%)	5 (1%)
Albumin			
Median (IQR)	39 (34 - 42)	39 (34 - 42)	38 (34 - 42)
Missing	4 (1%)	1 (<1%)	3 (1%)
Creatinine	73 (62 - 86)	73 (62 - 87)	72 (61 - 86)
PD-L1 tumour-infiltrating immune cell expression	0.90 (0.10 - 3.00)	0.90 (0.10 - 3.00)	0.90 (0.10 - 3.00)
PD-L1 tumour cell expression	0 (0 - 3)	0 (0 - 5)	0 (0 - 1)
Platelet levels	301 (241 - 382)	302 (242 - 377)	300 (240 - 383)
Neutrophil-to-lymphocyte ratio	4.2 (2.8 - 6.4)	4.3 (2.9 - 6.5)	4.2 (2.8 - 6.4)
Tobacco history			
Current	211 (31%)	108 (31%)	103 (30%)
Former	416 (61%)	203 (59%)	213 (63%)
Never	55 (8%)	32 (9%)	23 (7%)
Missing	1 (<1%)	0 (0%)	1 (<1%)
Liver metastasis	140 (20%)	73 (21%)	67 (20%)
Bone metastasis	185 (27%)	97 (28%)	88 (26%)
Genetic alteration in EGFR and ALK			
Negative_or_missing	682 (100%)	342 (100%)	340 (100%)
Positive	1 (<1%)	1 (<1%)	0 (0%)
NCI comorbidity score			
Median (IQR)	1.2 (0.0 - 1.7)	1.3 (0.0 - 1.7)	1.2 (0.0 - 1.7)
Missing	29 (4.2%)	11 (3.2%)	18 (5.3%)
CNS metastases at baseline	1 (<1%)	0 (0%)	1 (<1%)

Data are median (IQR) or number of patients (%).

IMpower132 ITT population

	Total No. 578	Atezolizumab No. 292	Control No. 286
Age	63 (56 - 69)	64 (57 - 70)	63 (56 - 68)
Sex			
Female	194 (34%)	100 (34%)	94 (33%)
Male	384 (66%)	192 (66%)	192 (67%)
ECOG performance status group			
0	240 (42%)	126 (43%)	114 (40%)
1+	336 (58%)	166 (57%)	170 (59%)
Missing	2 (<1%)	0 (0%)	2 (1%)
Metastasis site count	3 (2 - 4)	3 (2 - 4)	3 (2 - 3)
Haemoglobin			
Median (IQR)	131 (119 - 143)	131 (119 - 144)	132 (119 - 142)
Missing	1 (<1%)	0 (0%)	1 (<1%)
Lactate dehydrogenase			
Median (IQR)	234 (183 - 352)	242 (186 - 347)	229 (179 - 362)
Missing	21 (4%)	10 (3%)	11 (4%)
Albumin			
Median (IQR)	39 (35 - 42)	39 (35 - 42)	39 (35 - 43)
Missing	5 (1%)	1 (<1%)	4 (1%)
Creatinine			
Median (IQR)	70 (57 - 83)	70 (57 - 82)	70 (57 - 83)
Missing	1 (<1%)	0 (0%)	1 (<1%)
Platelet levels			
Median (IQR)	304 (247 - 398)	304 (249 - 397)	303 (244 - 400)
Missing	1 (<1%)	0 (0%)	1 (<1%)
Neutrophil-to-lymphocyte ratio			
Median (IQR)	3.9 (2.6 - 6.2)	3.8 (2.6 - 6.0)	3.9 (2.7 - 6.3)
Missing	2 (0.3%)	1 (0.3%)	1 (0.3%)
Tobacco history			
Current	127 (22%)	61 (21%)	66 (23%)
Former	384 (66%)	194 (66%)	190 (66%)
Never	67 (12%)	37 (13%)	30 (10%)
Liver metastasis	73 (13%)	37 (13%)	36 (13%)
Bone metastasis	148 (26%)	67 (23%)	81 (28%)
Genetic alteration in EGFR and ALK			
Negative_or_missing	571 (99%)	289 (99%)	282 (99%)
Positive	7 (1%)	3 (1%)	4 (1%)
NCI comorbidity score			
Median (IQR)	0 (0 - 2)	0 (0 - 2)	0 (0 - 1)
Missing	30 (5%)	18 (6%)	12 (4%)
CNS metastases at baseline	39 (7%)	14 (5%)	25 (9%)

Data are median (IQR) or number of patients (%).

IMmotion151 ITT population

	Total No. 915	Atezolizumab No. 454	Control No. 461
Age	61 (55 - 68)	62 (56 - 69)	60 (54 - 66)
Sex			
Female	246 (27%)	137 (30%)	109 (24%)
Male	669 (73%)	317 (70%)	352 (76%)
Metastasis site count	2 (1 - 2)	2 (1 - 2)	2 (1 - 2)
Karnofsky performance scale			
60	3 (<1%)	0 (0%)	3 (1%)
70	72 (8%)	40 (9%)	32 (7%)
80	141 (15%)	62 (14%)	79 (17%)
90	329 (36%)	180 (40%)	149 (32%)
100	370 (40%)	172 (38%)	198 (43%)
Histology			
Clear cell	845 (92%)	420 (93%)	425 (92%)
Sarcomatoid	26 (3%)	12 (3%)	14 (3%)
Other	44 (5%)	22 (5%)	22 (5%)
Memorial Sloan Kettering Cancer Cent/Motzer score			
0	179 (20%)	89 (20%)	90 (20%)
1	364 (40%)	182 (40%)	182 (39%)
2	265 (29%)	129 (28%)	136 (30%)
3	89 (10%)	47 (10%)	42 (9%)
4	18 (2%)	7 (2%)	11 (2%)
Liver metastasis	169 (18%)	85 (19%)	84 (18%)
Corrected Calcium			
Median (IQR)	2.4 (2.3 - 2.4)	2.4 (2.3 - 2.4)	2.3 (2.3 - 2.4)
Missing	18 (2.0%)	10 (2.2%)	8 (1.7%)
Haemoglobin	131 (117 - 144)	131 (118 - 144)	132 (116 - 144)
Platelet levels			
Median (IQR)	260 (208 - 330)	259 (208 - 323)	261 (208 - 332)
Missing	1 (<1%)	0 (0%)	1 (<1%)
Neutrophil			
Median (IQR)	4.5 (3.6 - 5.8)	4.6 (3.7 - 5.9)	4.5 (3.5 - 5.7)
Missing	8 (0.9%)	3 (0.7%)	5 (1.1%)
Lactate dehydrogenase			
Median (IQR)	195 (160 - 298)	193 (159 - 303)	197 (162 - 291)
Missing	4 (<1%)	2 (<1%)	2 (<1%)
Alkaline phosphatase			
Median (IQR)	93 (74 - 125)	94 (74 - 125)	93 (73 - 128)
Missing	1 (<1%)	0 (0%)	1 (<1%)
C-reactive protein			
Median (IQR)	7.2 (2.2 - 31.0)	6.8 (2.0 - 29.5)	7.5 (2.2 - 31.1)
Missing	52 (5.7%)	25 (5.5%)	27 (5.9%)
Tumour PD-L1 expression score	0.50 (0.00 - 1.00)	0.50 (0.00 - 1.00)	0.50 (0.00 - 2.00)
NCI comorbidity score			
Median (IQR)	0 (0 - 1)	0 (0 - 1)	0 (0 - 1)
Missing	51 (6%)	20 (4%)	31 (7%)

Data are median (IQR) or number of patients (%).

IMvigor211 ITT population

	Total No. 931	Atezolizumab No. 467	Control No. 464
Age	67 (60 - 73)	67 (59 - 73)	67 (61 - 73)
Sex			
Female	214 (23%)	110 (24%)	104 (22%)
Male	717 (77%)	357 (76%)	360 (78%)
Metastasis site count	2 (1 - 3)	2 (1 - 3)	2 (1 - 3)
PD-L1 tumor-infiltrating immune cell expression group			
IC0	306 (33%)	151 (32%)	155 (33%)
IC1	391 (42%)	200 (43%)	191 (41%)
IC2	234 (25%)	116 (25%)	118 (25%)
PD-L1 tumour cell expression group			
TC0	678 (73%)	341 (73%)	337 (73%)
TC1	95 (10%)	50 (11%)	45 (10%)
TC2	124 (13%)	64 (14%)	60 (13%)
TC3	34 (4%)	12 (3%)	22 (5%)
Albumin			
Median (IQR)	39 (36 - 43)	40 (36 - 43)	39 (36 - 42)
Missing	4 (<1%)	2 (<1%)	2 (<1%)
Creatinine			
Median (IQR)	101 (84 - 122)	100 (84 - 121)	103 (84 - 124)
Missing	1 (<1%)	1 (<1%)	0 (0%)
Platelet levels	244 (198 - 313)	244 (195 - 306)	246 (200 - 318)
Lactate dehydrogenase			
Median (IQR)	221 (176 - 328)	221 (177 - 328)	222 (175 - 329)
Missing	19 (2%)	11 (2%)	8 (2%)
Tobacco history			
Current	120 (13%)	60 (13%)	60 (13%)
Former	546 (59%)	266 (57%)	280 (60%)
Never	262 (28%)	140 (30%)	122 (26%)
Missing	3 (<1%)	1 (<1%)	2 (<1%)
Haemoglobin	119 (107 - 131)	119 (108 - 131)	119 (107 - 130)
Neutrophil-to-lymphocyte ratio			
Median (IQR)	3.5 (2.4 - 5.5)	3.5 (2.5 - 5.5)	3.6 (2.4 - 5.5)
Missing	4 (0.4%)	3 (0.6%)	1 (0.2%)
Lymphocyte-to-monocyte ratio			
Median (IQR)	2.2 (1.5 - 3.2)	2.2 (1.5 - 3.0)	2.2 (1.5 - 3.3)
Missing	4 (0.4%)	3 (0.6%)	1 (0.2%)
PLR			
Median (IQR)	183 (129 - 268)	181 (130 - 262)	184 (128 - 272)
Missing	4 (<1%)	3 (1%)	1 (<1%)
Liver metastasis	268 (29%)	138 (30%)	130 (28%)
Lung tumor site	397 (43%)	185 (40%)	212 (46%)
Bone metastasis	219 (24%)	110 (24%)	109 (23%)
Lymph node tumor site	542 (58%)	266 (57%)	276 (59%)
Visceral metastasis	612 (66%)	299 (64%)	313 (67%)
C-reactive protein			
Median (IQR)	15 (5 - 42)	15 (5 - 43)	15 (5 - 41)
Missing	63 (7%)	29 (6%)	34 (7%)
NCI comorbidity score			
Median (IQR)	0 (0 - 1)	0 (0 - 1)	0 (0 - 1)
Missing	22 (2%)	10 (2%)	12 (3%)
ECOG performance status			
0	424 (46%)	218 (47%)	206 (44%)
1	507 (54%)	249 (53%)	258 (56%)

Data are median (IQR) or number of patients (%).

Table S6: *Apparent and cross-validated Harrell's concordance index (C-index). Apparent C-index was derived from the fitted prognostic models in each RCT, whereas the cross-validated C-index was derived from the cross-validation evaluation of the prognostic models. The difference between these two indexes indicates potential degree of overfitting, with a larger positive difference indicating a likely greater degree of overfitting.*

RCT	Cross-validated C-index	Apparent C-index	Apparent minus cross-validated
OAK	0.70	0.76	0.06
IMpower130	0.64	0.76	0.12
IMpower150	0.66	0.77	0.11
IMpower133	0.62	0.76	0.14
IMbrave150	0.63	0.74	0.11
IMspire150	0.65	0.79	0.14
IMvigor211	0.69	0.77	0.08
IMmotion151	0.79	0.84	0.06
IMpower131	0.64	0.76	0.12
IMpower132	0.67	0.74	0.07

Table S7*. Bootstrapped variable importance for the top-ranked variables in IMvigor211, including those with bootstrapped rank confidence intervals within 10 and a best rank of 1.*

Variable	Median rank	Bootstrapped 95% CI	Best rank	Worst rank
Albumin	1	1-2	1	7
C-reactive protein	2	1-3	1	9
Lactate dehydrogenase	3	2-3	1	7
Haemoglobin	5	4-6	1	13
Liver metastasis status	5	4-6	1	17
Neutrophil-to-lymphocyte ratio	6	5-8	1	15
Lymphocyte-to-monocyte ratio	6	4-7	1	16

Supplementary Figures

***Figure S1**: Distribution of the risk score predicted by the prognostic model in the ten RCTs. Risk scores are scaled to a maximum of one and minimum of zero. Median:Mean denotes median-to-mean ratio.*

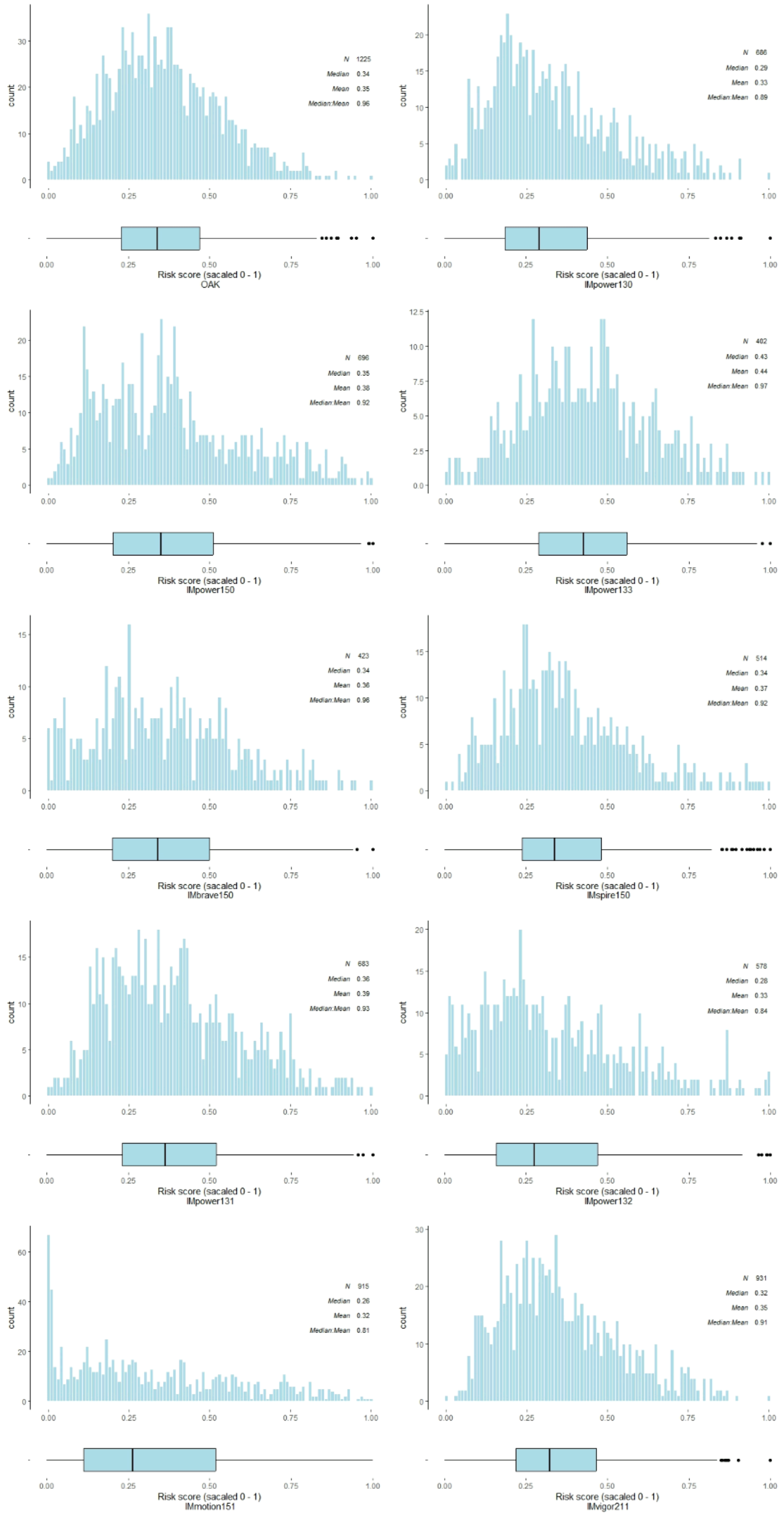

***Figure S2**: Calibration plots derived from XGBoost prognostic models predicted at 24 months during the cross-validation model evaluation.*

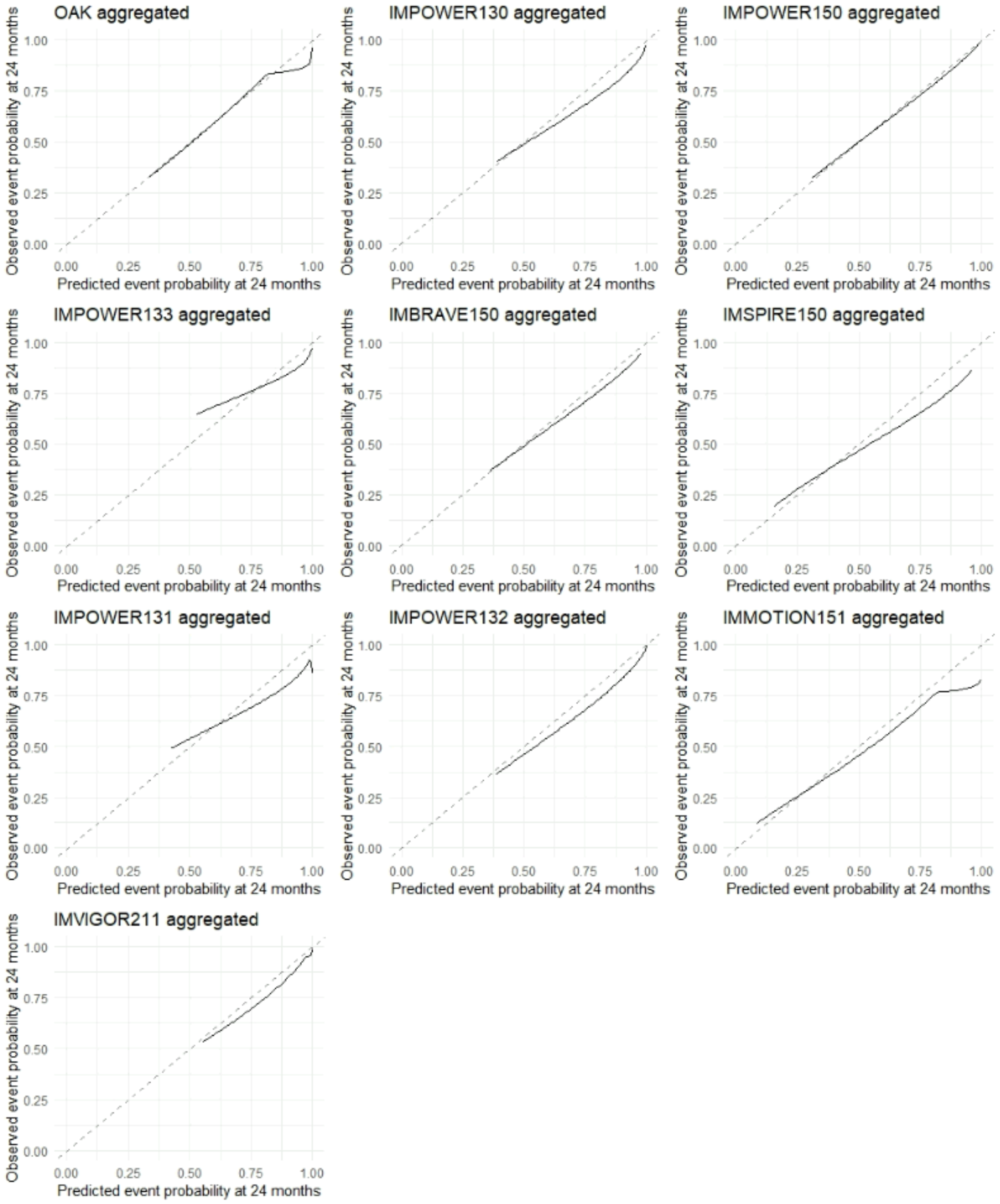

***Figure S3**: Line plots of absolute treatment effect quantified by the difference of restricted mean survival times (RMSTs) between control arm and atezolizumab arm evaluated at 24 months. A negative RMST difference represents atezolizumab treatment benefit.*

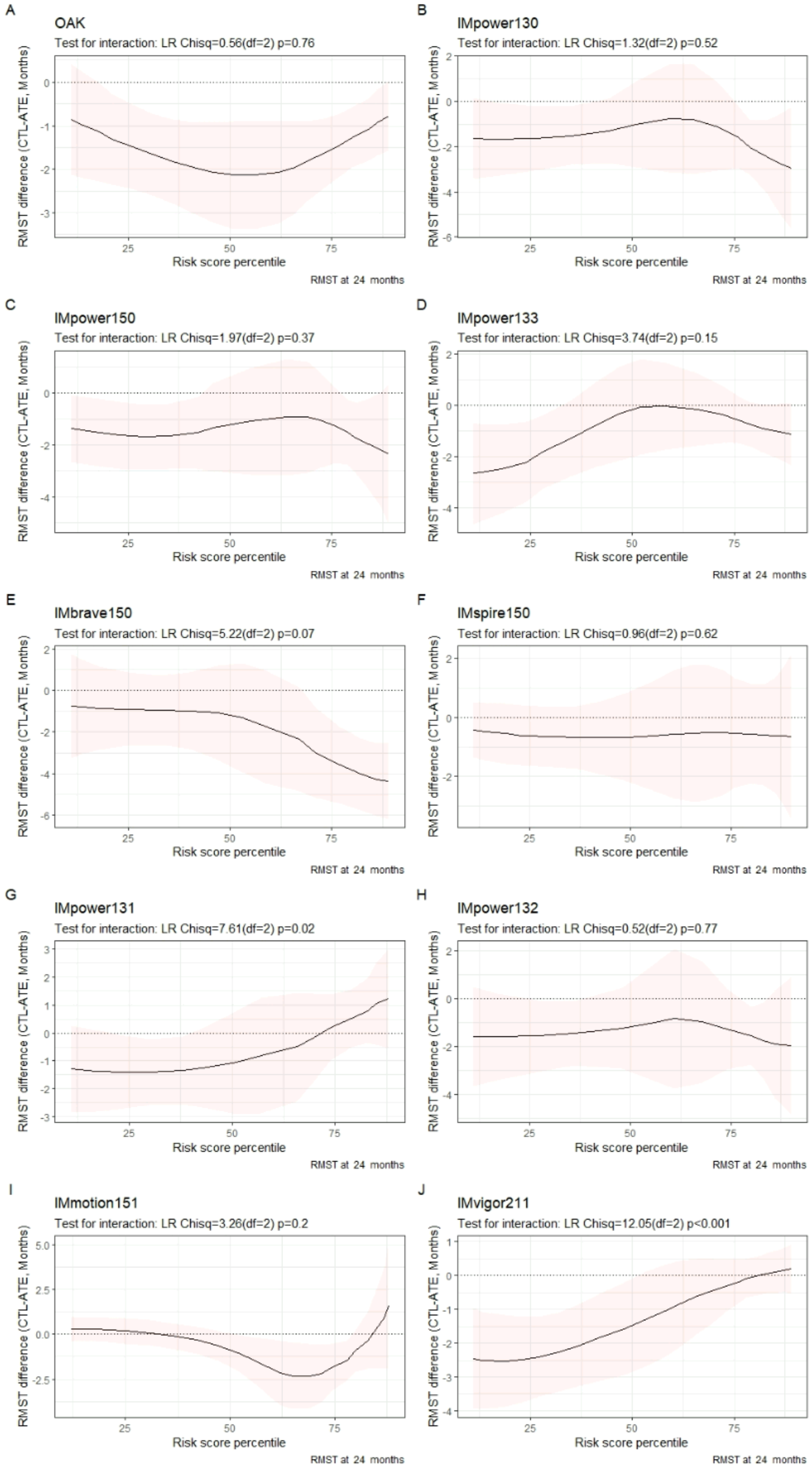

Figure S4: *Forest plot and line plot from two-stage multivariate meta-analyses of the five NSCLC trials displaying the aggregated risk group treatment efficacy (**top, forest plot**) and aggregated treatment efficacy relative to that at the median risk score (**bottom, line plot**). The five RCTs included OAK, IMpower130, IMpower131, IMpower132, and IMpower150. In addition to p-values, a lack of treatment effect heterogeneity is also indicated by the wide confidence intervals spanning across the line of equivocal efficacy (HR=1) in the line plot.*

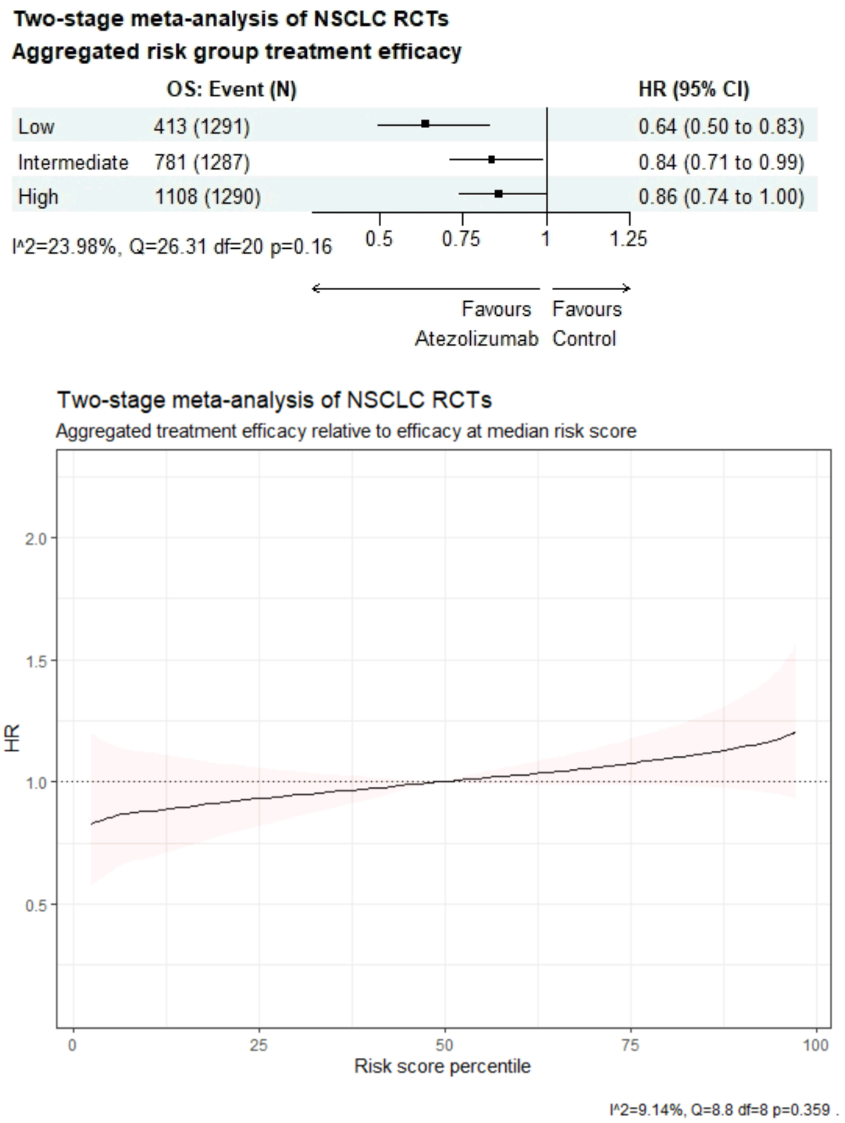

Figure S5*: Overall survival in Kaplan-Meier plots and hazard ratios (HRs) in forest plots for the high, intermediate, and low risk groups defined by Memorial Sloan Kettering Cancer Centre (MSKCC) staging system for RCC in IMmotion151. Solid lines: Atezolizumab-based treatment arms. Dashed lines: control arms. Red: Low risk group. Yellow: intermediate risk group. Blue: high risk group. CI: Confidence interval.*

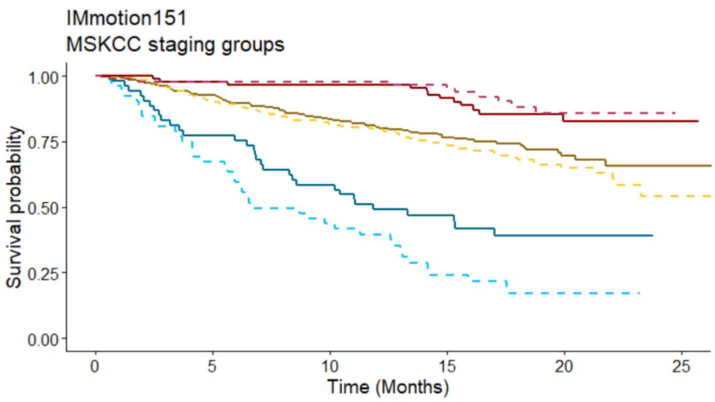

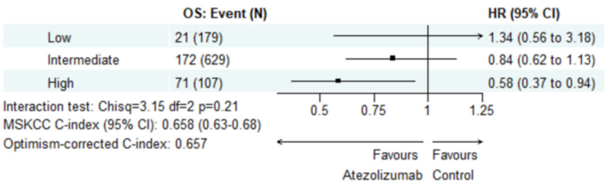

Figure S6*: Overall survival in Kaplan-Meier plots and hazard ratios (HRs) in forest plots for the three groups defined by the programmed death-ligand 1 (PD-L1) expression on tumour-infiltrating immune cells (IC groups: IC0, IC1, IC2) in IMvigor211. Solid lines: Atezolizumab-based treatment arms. Dashed lines: control arms. Red: IC2 group. Yellow: IC1 group. Blue: IC0 group. CI: Confidence interval.*

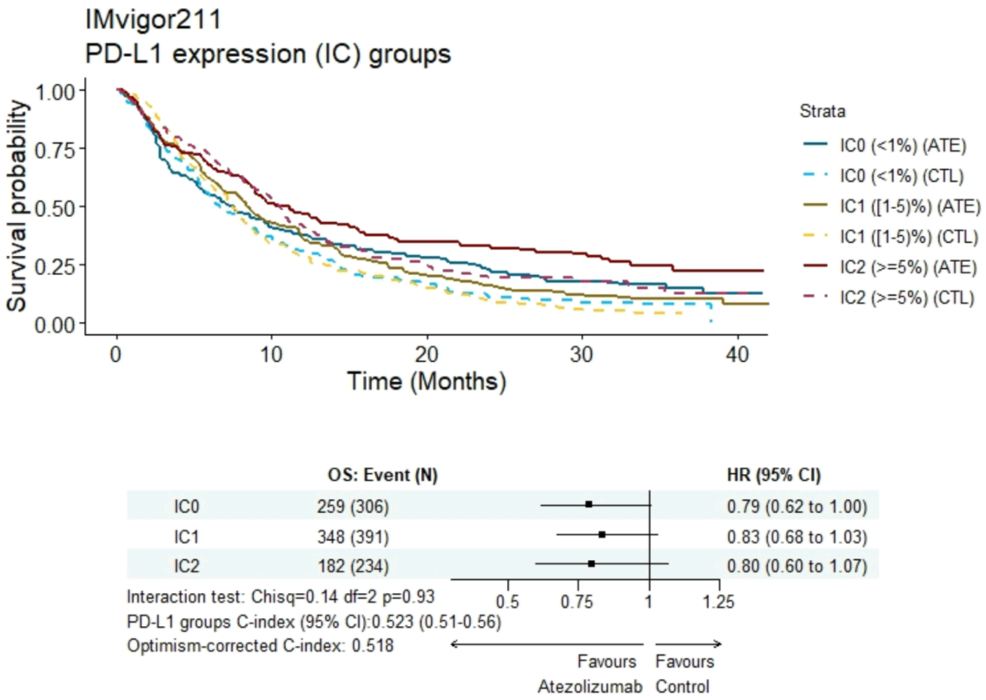

Supplement: pkaf127_Supplementary_Data [file pkaf127_supplementary_data.pdf]
